# Rapid electrochemical nitrate sensing using flexible screen-printed electrodes modified with carbon black/poly (1,5-diaminonaphthalene)/copper dendrite hybrid interfaces

**DOI:** 10.1039/d6ra02725c

**Published:** 2026-07-06

**Authors:** Saad Benhaiba, Anas El Attar, Abdelaziz Elgamouz, Charafeddine Jama, Amine Ezzahi, Mama El Rhazi

**Affiliations:** a Laboratory of Materials Membranes and Environment, Faculty of Sciences and Technologies, University Hassan II Casablanca BP 146 20650 Mohammedia Morocco mama.elrhazi@fstm.ac.ma +212 523315353 +212 523315352; b Applied Chemistry Research Group, Department of Chemistry, College of Sciences, University of Sharjah P.O. Box 27272 Sharjah United Arab Emirates; c University Lille, CNRS, INRAE, Centrale Lille, UMR 8207-UMET-Unité Matériaux Et Transformations Lille 59000 France

## Abstract

The rising demand for sensitive and accurate nitrate ion detection in water has motivated the design of novel electroactive sensing materials. Conducting polymers, notably, provide a highly adaptable surface for engineering materials with superior electrochemical performance. In this study, a composite electrode for nitrate detection was fabricated by polymerizing a 1,5-diaminonaphthalene (poly 1,5-DAN) layer on a carbon black modified screen-printed electrode (CB/SPE), followed by the electrodeposition of copper particles through cyclic voltammetry. The resulting electrodes were characterized by scanning electron microscopy (SEM) to examine surface morphology revealing a dendritic form of Cu (Cu Ds) and by X-ray photoelectron spectroscopy (XPS) to determine their chemical composition. Electrochemical techniques were employed throughout the study to investigate the electrode behavior and evaluate the nitrate sensing performance. The Cu Ds/poly 1,5-DAN/CB/SPE electrode showed strong performance for nitrate sensing, offering a broad linear detection range from 1 to 175 µM and a low detection limit of 0.28 µM. In addition, the sensor maintained good stability throughout its measurements and a high reproducibility, confirming its robustness for reliable analytical use. Notably, the simple deposition method used to assemble the polymer, carbon black and copper particles produces a functional composite material with potential applications not only in nitrate sensing but also in water quality monitoring and denitrification.

## Abstract 2

Copper dendrites and poly 1,5-diaminonaphthalene synergistically enhance the electrochemical sensing of nitrate ions at carbon black-based screen-printed electrodes.

## Graphical abstract

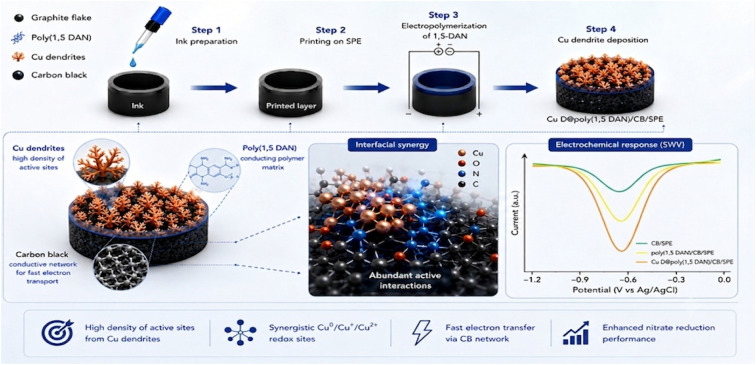

## 1 Introduction

Nitrate contamination represents a major environmental challenge despite the essential role of nitrates in agriculture and food production. Excessive nitrate accumulation in water resources, largely driven by intensive fertilizer use and industrial activities, poses significant risks to ecosystems and human health.^1–3^ Recent reviews document the global scale of nitrate contamination and emphasize the accelerating trend in agricultural regions, linking fertilizer intensification and land-use change to rising groundwater nitrate levels.^4^ Nitrate (NO_3_^−^) can be transformed into nitrite (NO_2_^−^), then into nitric oxide (NO), and under certain conditions leads to the formation of N-nitrosamines that can result in the formation of several toxic nitrogen compounds,^5^ which are linked to liver disease and stomach cancer.^6^ Nitrate's danger is most starkly seen in infants, where it can cause “blue baby syndrome”,^7^ a serious condition that prevents their blood from carrying enough oxygen. In response to this and other severe health risks, according to the World Health Organization, the amount of nitrate in drinking water should not exceed 50 mg L^−1^ to ensure it remains safe for consumption.^8^ Consequently, the development of sensitive, reliable and rapid nitrate monitoring technologies has become important for environmental protection and public safety. Several analytical methods have been developed for nitrate determination in water, including ultraviolet (UV) spectrometry,^9^ ion chromatography,^10^ along with other approaches such as flow injection analysis, capillary electrophoresis, chemiluminescence detection, high-performance liquid chromatography (HPLC), and gas chromatography-mass spectrometry (GC-MS).^10–12^ While these methods offer high sensitivity and specificity, their widespread application is often limited by the requirement for sophisticated instrumentation, high operating costs and skilled personnel.^13^ These limitations have stimulated growing interest in electrochemical sensing technologies which provide a promising alternative due to their simplicity, rapid response, high selectivity and portability.^14–18^ In this context, considerable efforts have been devoted to enhancing the performance of electrochemical nitrate sensors through the development of efficient electrocatalytic materials.

A range of metals, such as Cu, Ag, Ni, Pt and Au, has been utilized for electrode modification, serving as catalysts for the electroreduction of NO_3_^−^. The modification of the electrode surface increase both the efficiency and performance of the electrochemical reduction reaction.^19–22^ Among various electrocatalytic metals, copper (Cu) stands out for the electrocatalytic conversion of nitrate to nitrites due to cost-effectiveness, favorable electrical properties, biocompatibility, and large specific surface area.^23,24^ Moreover, designing hybrid electrocatalytic interfaces through the combination of copper nanostructures with conductive carbon supports has proven to be a powerful strategy for improving nitrate electroreduction. Carbon nanostructure not only enhances electron transport but also provides a porous and high-surface-area framework that promotes the uniform dispersion of catalytic copper species.^25^ Among carbon-based nanomaterials, carbon black (CB) nanoparticles stand out due to their exceptionally low cost (approximately $1 per kilogram).^26^ This affordability makes them particularly attractive for the development of cost-effective sensor technologies.^18,20^ Moreover, they are well-known for their large surface area and their ability to promote electronic transfer.^27,28^ In this context, Anurag and co-workers created a nitrate sensor by combining graphene oxide with multiwalled carbon nanotubes, and subsequently improving the composite through the electrochemical deposition of copper nanoparticles onto a screen-printed electrode. This hybrid structure provided strong electrocatalytic activity toward nitrate, allowing the device to detect the ion at very low concentrations, with a reported detection limit of 3.3 µM.^29^ In another work, Li *et al.* introduced a nitrate sensor built on carbon nanofibers that were simultaneously modified with copper, demonstrating an effective strategy for integrating copper nanostructures with carbon materials to enhance electrochemical nitrate detection.^30^ Mirabootalebi *et al.*^31^ optimized copper morphology on carbon based screen-printed electrodes (SPEs) using machine-learning-assisted pulse electrodeposition, demonstrating that the catalytic performance of Cu toward nitrate reduction is strongly governed by its morphology, surface roughness, and active-site density, while Farina *et al.*^32^ reported Cu microflower structures whose abundant edges, defects, and high surface area significantly enhanced morphology-driven sensitivity by increasing the number of accessible catalytic sites for nitrate reduction. Wei *et al.*^33^ employed Cu-modified boron-doped diamond (BDD) electrodes, where Cu served as the primary electrocatalyst while the BDD substrate provided excellent chemical stability, antifouling properties, and a wide potential window, resulting in a broad detection range and long-term operational stability. More recently, Ankalgi *et al.*^34^ introduced MXene-supported Cu–Ag nanohybrids, in which Cu acted as the principal nitrate-reduction catalyst, Ag improved electron-transfer kinetics and selectivity, and the highly conductive two-dimensional MXene support enhanced nanoparticle dispersion and charge transport through a synergistic bimetallic architecture. Furthermore, polymeric matrices serve as multifunctional supports, ensuring structural cohesion and homogeneous distribution of the active components while preserving their accessibility. Such synergistic interactions between the metallic catalyst, carbon nanostructure, and polymer support are key to achieving superior catalytic efficiency and sensing performance.^35,36^ This approach demonstrates an optimized layering of materials to enhance overall conductivity and functionality. Essousi *et al.* achieved a notable improvement in the limit of detection by employing a sensor constructed from ion-imprinted polyaniline coated with copper nanoparticles.^37^ The enhanced conductivity of the polyaniline layer significantly facilitated electron transfer on the sensor's surface, guiding to a more pronounced and reliable nitrate detection signal, whereas Amini *et al.* developed a NO_3_^−^ sensor with a LOD of 2.1 µM, utilizing a Cu/TiO_2_ core–shell structure with Nafion and polyalizarin immobilized on the electrode.^38^ While the sensor demonstrates notable sensitivity, its preparation involves a lengthy and complex process. Motaghedifard *et al.* explored the integration of copper with conducting polymers, specifically utilizing polyaniline as a substrate before the deposition of copper and Au/MWCNT.^39^ Although significant progress has been achieved in the development of copper-based nitrate sensors, several challenges remain, particularly regarding electrode stability and the time-consuming preparation procedures. To overcome these limitations, we developed a hybrid electrochemical platform combining copper dendrites, poly(1,5-diaminonaphthalene) (PDAN), and carbon black. The amine-rich PDAN matrix provides efficient anchoring sites for copper deposition and promotes the formation of stable and well-dispersed nanostructures.^40,41^ While carbon black enhances electron transfer and increases the electroactive surface area, the synergistic combination of these components is expected to improve both the sensitivity and stability of nitrate sensing.

Herein, a rapid and straightforward strategy, was developed for the fabrication of a Cu dendrites/poly 1,5-diaminonaphthalene/carbon black modified screen-printed electrode (Cu Ds/poly 1,5-DAN/CB/SPE) toward highly sensitive nitrate sensing. The fabrication procedure involved the sequential drop-casting of a carbon black suspension onto the SPE surface, electropolymerization of 1,5-diaminonaphthalene, and subsequent electrochemical growth of Cu dendrites by cyclic voltammetry. The proposed architecture combines the high conductivity of CB, the electroactive and stable interface provided by the conducting polymer, and the large active surface area of Cu dendrites, leading to a synergistic enhancement of the electrochemical performance. Notably, the entire fabrication process was completed within approximately 40 min, highlighting the practical potential of the approach for rapid and scalable sensor production. The physicochemical properties and surface morphology of the prepared electrode were comprehensively characterized using XPS, XRD, and SEM–EDX analyses, while its electrochemical and interfacial behaviors were investigated by cyclic voltammetry and electrochemical impedance spectroscopy. The electrocatalytic activity of the Cu Ds/poly 1,5-DAN/CB/SPE toward nitrate reduction was evaluated by square-wave voltammetry, revealing a well-defined reduction response over a broad linear range from 1 to 175 µM with a low detection limit. Furthermore, the sensor exhibited excellent selectivity, repeatability, reproducibility, and long-term stability, and its applicability was successfully demonstrated in real-sample analysis.

## 2 Materials and methods

### 2.1 Chemicals and reagents

Screen printed electrodes (ItalSens IS-C) were obtained from Palmsens. 1,5-diaminonaphthalene (1,5-DAN), potassium ferricyanide [K_3_Fe(CN)_6,_ ACS reagent >99%], potassium ferrocyanide/[K_4_Fe(CN)_6_·3H_2_O; ACS reagent >99%], copper(ii) sulfate pentahydrate (CuSO_4_·5H_2_O) and carbon black were purchased from Loba Chemie. Sodium sulfate (Na_2_SO_4_), and potassium nitrate (KNO_3_) were obtained from Sigma-Aldrich. Hydrochloric acid (HCl, 37%) was supplied by Scharlab. All analytical solutions were prepared using double-distilled water.

### 2.2 Materials and apparatus

Fourier transform infrared (FTIR) spectroscopy was carried out using an Shimadzu Affinity-1S FTIR Spectrometer equipped with a Golden Gate single-reflection attenuated total reflectance (ATR) accessory. Spectra were recorded over the wavenumber range of 500–4000 cm^−1^ with a resolution of 16 cm^−1^ in order to analyze the structural characteristics of CB/SPE, poly 1,5-DAN/CB/SPE, and Cu/poly 1,5-DAN/CB/SPE materials. The surface morphology of the prepared electrocatalysts was investigated using scanning electron microscopy (SEM) coupled with energy-dispersive X-ray spectroscopy (EDX) using a Zeiss SUPRA 55-VP Scanning Electron Microscope. Crystallographic information and phase identification of copper supported on Poly 1,5-DAN/CB/SPE were obtained using an PANalytical X'Pert PRO MPD X-ray Diffractometer. X-ray photoelectron spectroscopy (XPS) analysis was performed with a Thermo Scientific Nexsa G2 XPS System using Al Kα radiation (1486.6 eV) under ultra-high vacuum conditions of approximately 10^−9^ mbar. High-resolution spectra were collected with a pass energy of 50 eV, while survey scans were acquired with a pass energy of 200 eV using a 400 µm analysis spot. Charge neutralization was achieved with a dual flood gun, and the binding energies were referenced to the C 1s peak at 284.8 eV for calibration.

### 2.3 Preparation of the modified electrodes

The screen-printed electrode was first coated by drop-casting three consecutive 2 µL portions of a carbon black suspension (1 mg mL^−1^ in DMF). After each application, the electrode was placed in an oven at 60 °C for 5 minutes to ensure proper drying and adhesion of the CB layer.

A poly(1,5-diaminonaphthalene) film was then generated electrochemically by cyclic voltammetry in a solution containing 5 mM 1,5-diaminonaphthalene and 0.1 M HCl, using a scan rate of 50 mV s^−1^. Copper dendrites were subsequently deposited onto the polymer-coated surface through cyclic voltammetry in an electrolyte composed of 0.1 M CuSO_4_·5H_2_O and 0.1 M H_2_SO_4_. The potential was cycled between −1.0 V and 0 V at a scan rate of 0.1 V s^−1^, following the procedure described by Mumtarin *et al.*^42^

### 2.4 Electrochemical measurements

Electrochemical experiments were conducted using a PalmSens4 Potentiostat controlled by PSTrace Software (version 5.9). The acquired electrochemical data were transmitted *via* Bluetooth and monitored on a smartphone interface. Before each measurement, the Cu Ds/poly 1,5-DAN/CB/SPE sensor was carefully rinsed with distilled water to remove any surface impurities and ensure consistent analytical performance. CV experiments were performed in a 0.1 M KCl electrolyte containing 5.0 mM Fe(CN)_6_]^3−/4−^. The potential was scanned from −0.2 to 0.6 V *versus* Ag/AgCl to evaluate the electrochemical behavior of the modified electrode. EIS measurements were carried out in the same solution at a fixed potential of 0.25 V *versus* Ag/AgCl with an AC amplitude of 10 mV, over a frequency range from 100 mHz to 100 kHz. Additional EIS experiments were conducted in 0.1 M sodium sulfate (Na_2_SO_4_, pH 5) containing 1 mM nitrate (NO_3_^−^), using an applied potential of −0.85 V *versus* Ag/AgCl across the same frequency range. Square wave voltammetry (SWV) was employed for nitrate detection in 0.1 M Na_2_SO_4_ (pH 5). Increasing concentrations of NO_3_^−^ were sequentially added to the blank electrolyte in order to construct calibration curves for quantitative analysis. All electrochemical measurements were carried out at room temperature.

### 2.5 Real sample analysis

To evaluate how well the developed method performs under practical conditions, the Cu Ds/poly 1,5-DAN/CB/SPE sensor was applied to the analysis of freshly collected well-water samples from the Mohammedia region in Morocco. All measurements were carried out using the optimized electrochemical parameters established earlier in the study. After analysis, recovery values and relative standard deviations were calculated to assess the method's accuracy and precision, providing a clear indication of its reliability for real-sample monitoring.

## 3 Results and discussion

### 3.1 The electropolymerization of 1,5-DAN

**Fig. 1:**
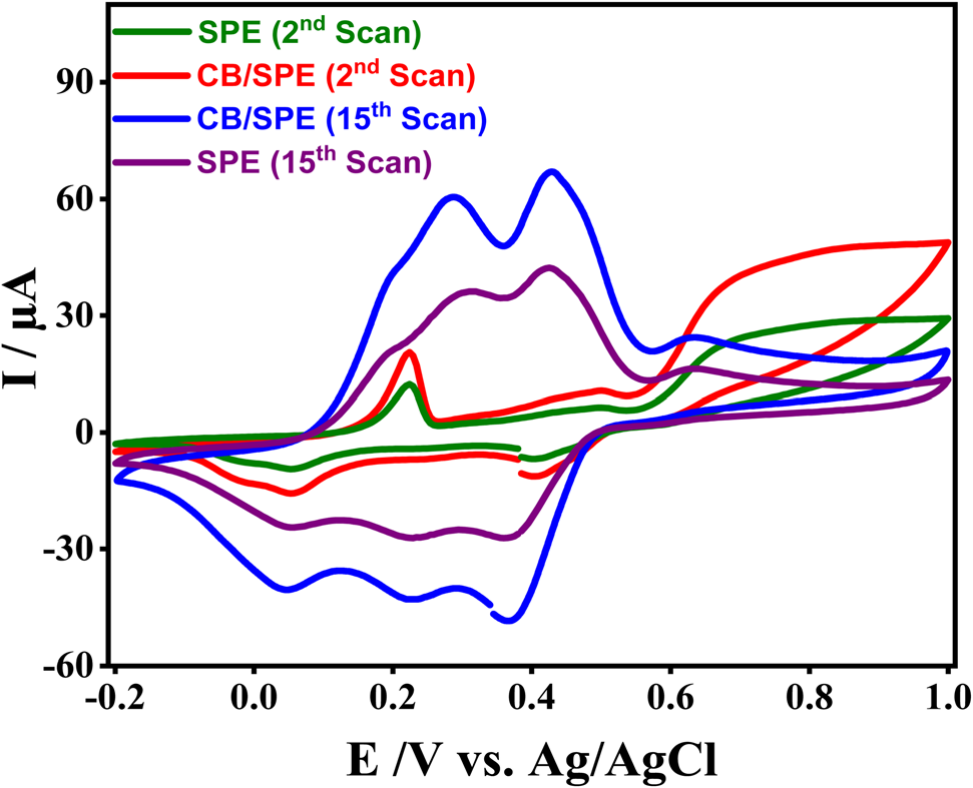
Cyclic voltammograms of 5 mM 1,5-DAN in 0.1 M HCl recorded at a scan rate of 50 mV s^−1^, showing the electropolymerization behavior after 2 and 15 cycles on both CB-modified SPE and the unmodified (bare) SPE.

Electropolymerization provides a controlled and efficient way to anchor polymer films onto conductive surfaces because the growth of the polymer can be finely tuned by adjusting the electrochemical conditions applied at the electrode. For 1,5-diaminonaphthalene (DAN), this process generally utilizes a standard three-electrode configuration incorporating a working electrode, a counter electrode, and a reference electrode. The working electrode is immersed in a solution that contains the DAN monomer along with an appropriate supporting electrolyte, allowing the polymer film to form directly on the electrode surface as the potential is cycled. The polymerization process is then initiated using techniques such as cyclic voltammetry or by applying potentiostatic or galvanostatic methods, depending on the experimental requirements.^40,43,44^ Fig. S1 (SI) presents the cyclic voltammograms recorded during the electropolymerization of 1,5-diaminonaphthalene (1,5-DAN) at both the screen-printed electrode (SPE) and the carbon black-modified screen-printed electrode (CB/SPE) in acidic medium. To elucidate the influence of the substrate and the number of potential cycles on film growth, a comparative study was conducted between the bare SPE and the CB/SPE, considering both the initial stage of polymerization (first two cycles) and extended cycling (15 cycles). During the first scan, a well-defined anodic peak appears around 0.7 V, corresponding to the oxidation of the monomer into its radical cation. When electropolymerization was limited to two consecutive scans (Fig. 1), the anodic peak currents recorded at the bare SPE reached 11.2 µA at 0.22 V and 6.1 µA at 0.50 V. In contrast, significantly higher currents of 20.5 µA at 0.22 V and 11.2 µA at 0.50 V were obtained at the CB/SPE under identical conditions. These comparatively low currents at this early stage indicate that the polymer film is still in its nucleation and initial growth phase. The enhanced response observed at the CB/SPE highlights the beneficial role of carbon black, which promotes electron transfer and improves the initial formation of the poly 1,5-DAN layer due to its high surface area and excellent electrical conductivity.

**Scheme 1:**
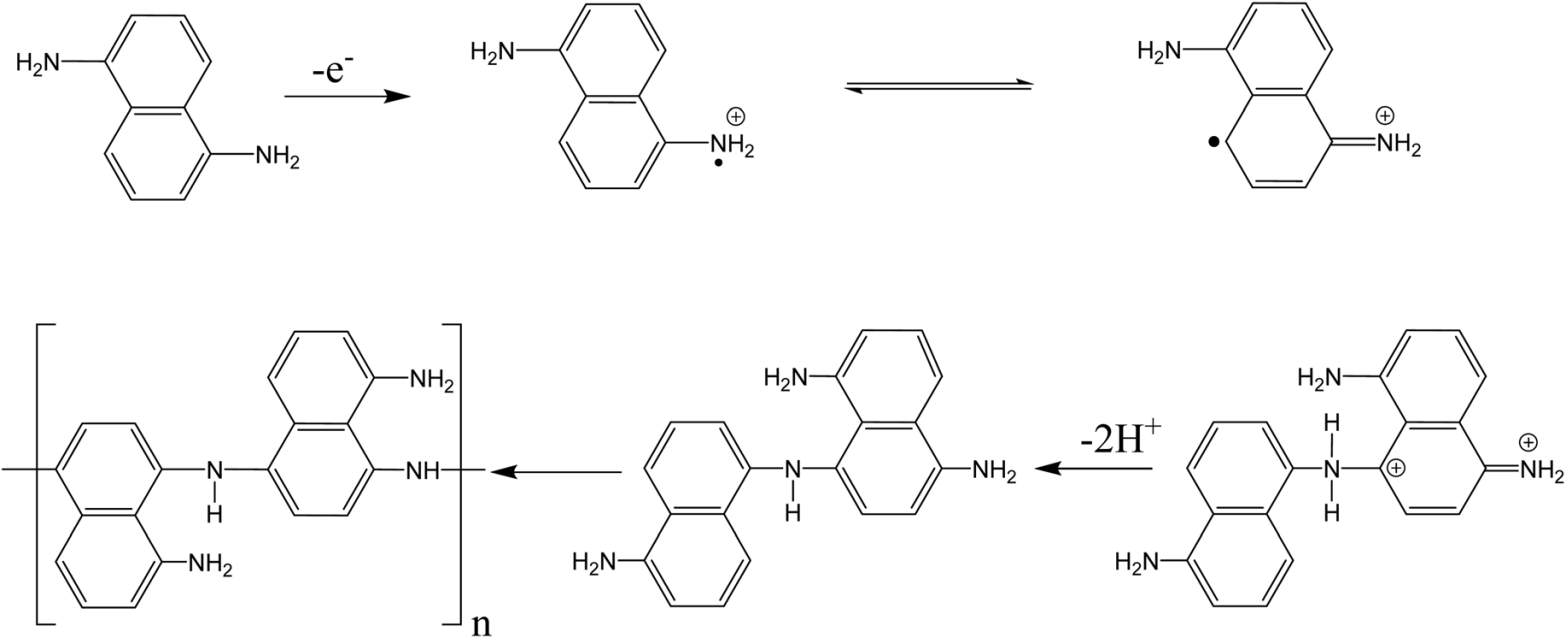
Electropolymerization mechanism of 1,5-diaminonaphthalene.

Subsequently, the electropolymerization process was then completed by performing 15 cyclic scans within a potential window from −0.2 to 1.0 V *versus* Ag/AgCl at a scan rate of 50 mV s^−1^ in an electrolyte composed of 0.1 M HCl and 5.0 mM 1,5-diaminonapththalene. As the cycling proceeded, two cathodic peaks (around 0.37 and 0.04 V) and two anodic peaks (around 0.25 and 0.43 V) gradually appeared. These peaks correspond to the redox transitions associated with the formation of the poly 1,5-DAN film on the electrode surface. The progressive increase in peak currents with each cycle indicates the continuous deposition of the electroactive polymer layer, confirming the successful growth of a conductive polymer film. After 15 cycles, the anodic peak currents at the CB/SPE reached 60.6 and 70.0 µA, respectively, whereas lower values of 35.8 and 42.8 µA were measured at the bare SPE under the same conditions. The electropolymerization of 1,5-DAN at the CB-modified SPE results in a current enhancement, attributable to the superior conductivity and high surface area of carbon black, which facilitates charge transfer and accelerates film growth, highlighting the synergistic effect of the conductive carbon framework. Similar improvements have been reported by Zhang *et al.* using multiwalled carbon nanotubes and in graphene-based polymer composites.^45^ The stable and reproducible redox features observed over consecutive scans confirm the formation of a robust and electroactive polymer layer, validating the successful synthesis of poly 1,5-DAN (Scheme 1).

### 3.2 Electrodeposition of the copper dendrites on poly1,5-DAN/CB/SPE

**Fig. 2:**
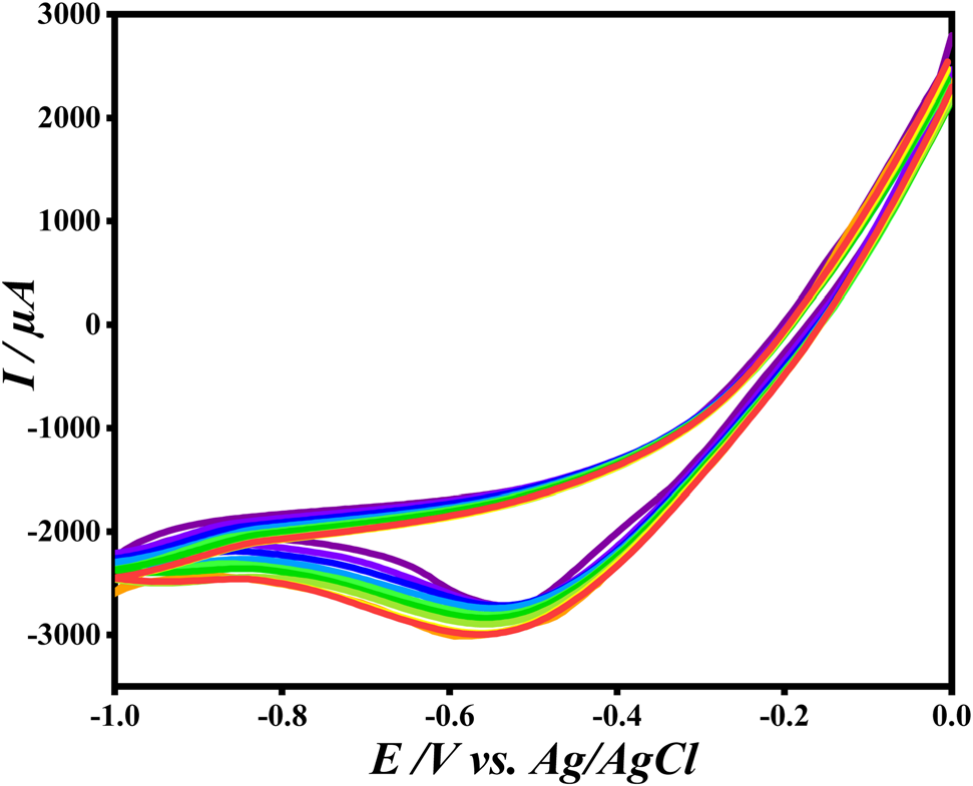
Cyclic voltammogram illustrating the electrodeposition of copper on the poly 1,5-DAN/CB/SPE in 0.1 M H_2_SO_4_ and 0.1 M CuSO_4_.

After forming the poly 1,5-DAN layer on both the bare SPE and the CB-modified SPE, the poly 1,5-DAN/CB/SPE was transferred into an electrolyte containing 0.1 M CuSO_4_ and 0.1 M H_2_SO_4_. Copper was then electrodeposited onto the surface by cyclic voltammetry, using the 10th scan recorded at a scan rate of 100 mV s^−1^ over a potential window ranging from 0 to −1.0 V *versus* Ag/AgCl. As shown in Fig. 2, the voltammograms display a well-defined cathodic peak at −0.55 V, characteristic of the reduction of Cu^2+^ to metallic copper, in agreement with previously reported values.^42,46,47^ For comparison, copper electrodeposition was also carried out on the CB/SPE, as presented in Fig. S2a and S2b (SI). The comparison clearly shows that the poly 1,5-DAN/CB/SPE exhibits significantly higher cathodic currents and a more pronounced reduction peak than the unmodified CB/SPE, indicating that the presence of the polymer ensures that the film of copper oxide is well dispersed on the surface and maintains uniform electrochemical properties across the electrode surface. Furthermore, the formation of a stable complex of copper film with poly 1,5-DAN/CB/SPE,^48–50^ which is essential for achieving reliable and reproducible sensor performance.

### 3.3 Characterization of electrode architecture and performance

**Fig. 3:**
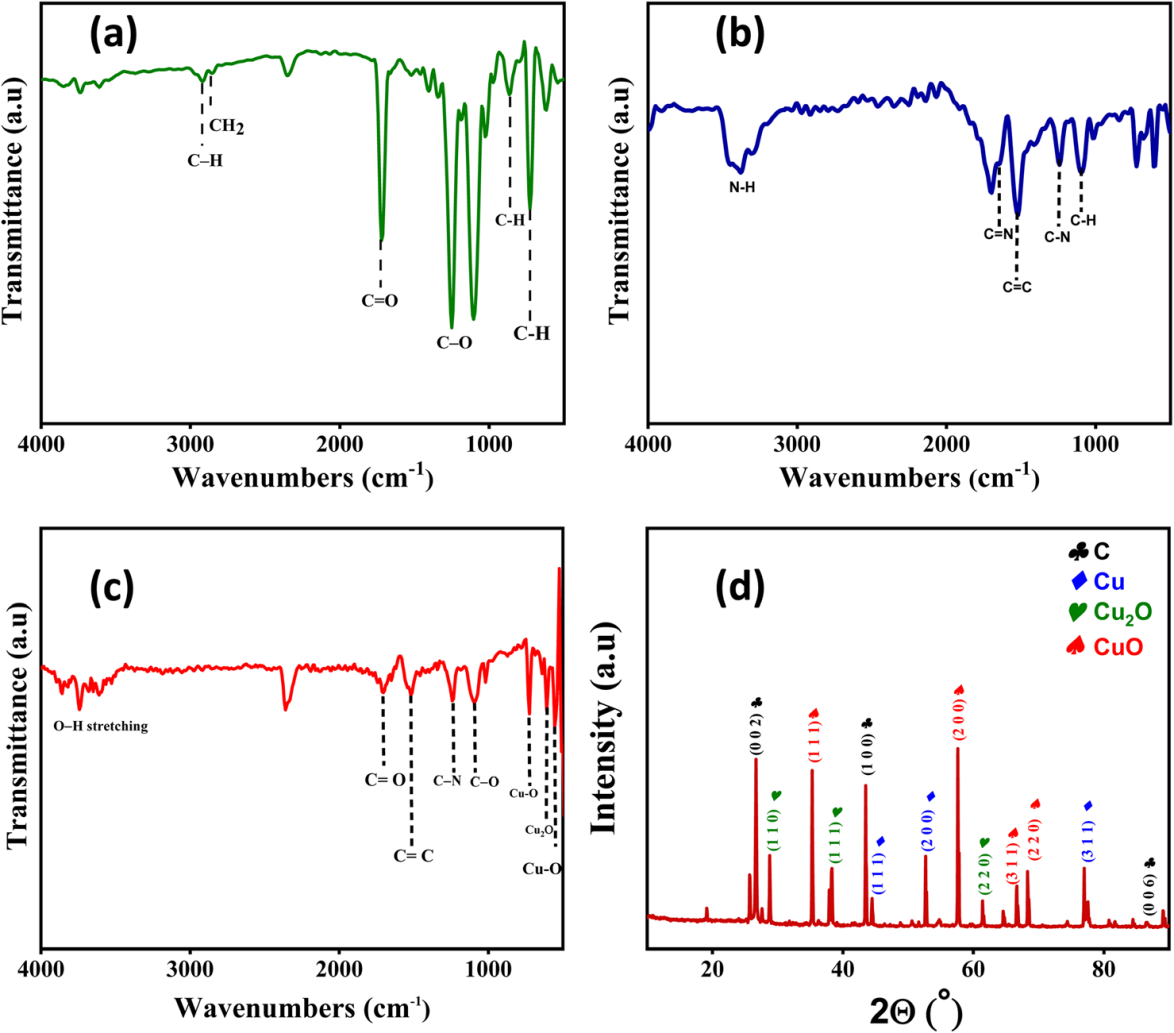
FTIR spectra of: (a) CB/SPE, (b) poly 1,5-DAN/CB/SPE, (c) Cu Ds/poly 1,5-DAN/CB/SPE, and (d) XRD analysis of Cu Ds/poly1,5-DAN/CB/SPE.

FTIR spectroscopy was used to examine the chemical features of the prepared electrodes CB/SPE, poly 1,5-DAN/CB/SPE, and Cu Ds/poly 1,5-DAN/CB/SPE. The corresponding spectra are shown in Fig. 3a–c, respectively. For the CB/SPE electrode, characteristic absorption bands appear in the 1650–1800 cm^−1^ region, which are attributed to C=O stretching vibrations, confirming the presence of carbonyl and carboxyl functional groups on the carbon black surface and the functionalization of the CB.^51^ A noticeable band near 1400 cm^−1^ is associated with carboxyl–carbonate structures and, reflecting the typical chemical signatures of carbon-based materials.^52^ Additionally, the absorption bands at 860 cm^−1^ and 720 cm^−1^ correspond to out-of-plane C–H deformation vibrations, which are characteristic of aromatic ring structures.^53^

For the poly 1,5-DAN/CB/SPE electrode, the FTIR spectrum exhibits characteristic peaks at 1584 cm^−1^ and 1640 cm^−1^, attributed to the C–C stretching of the naphthalene ring and the C=N stretching vibration of DAN. In addition, the band observed around 2930 cm^−1^ is attributed to C–H stretching vibrations associated with the aromatic ring, consistent with the findings reported by Arab *et al*.^54^ The N–H stretching vibration appears as a broad absorption band near 3400 cm^−1^, in agreement with the observations reported by Nguyen *et al.*^55^ Additionally, the peak observed at 1285 cm^−1^ corresponds to C–N stretching vibrations.^56^ Together, these spectral features confirm the successful formation of the poly 1,5-DAN film on the CB/SPE surface.

For the Cu Ds/poly 1,5-DAN/CB/SPE, the FTIR spectrum shows a distinct C–N stretching vibration appearing near 1200 cm^−1^. A pronounced band at 1685 cm^−1^ corresponds to C=O stretching, which is typically associated with oxidized DAN ring structures within the Cu/poly 1,5-DAN composite.^57^ Additional absorption bands at 555 cm^−1^ and 725 cm^−1^ correspond to Cu–O stretching modes, while the peak near 600 cm^−1^ provides clear evidence of copper oxide particles formed on the surface of the poly 1,5-DAN film.^58,59^ The main aromatic ring C=C stretching mode, observed at approximately 1515 cm^−1^, suggests coordination between copper and amine nitrogen atoms, thus confirming the formation of a metal–polymer complex.^60^ Overall, the FTIR analysis confirms the successful formation of the poly 1,5-DAN film and the deposition of copper dendrites on the modified electrode surfaces.

The crystalline structure of the Cu Ds/poly 1,5-DAN/CB/SPE was examined using XRD, and the corresponding XRD is presented in Fig. 3d. The characteristic broad diffraction peaks observed at 26.63° and 43.65° are attributed to the carbon black deposited on the SPE.^61^ Additionally, diffraction peaks appearing at approximately 28.75° and 61.5° are assigned to the crystalline planes of cuprous oxide (Cu_2_O), confirming the successful incorporation of copper oxide nanoparticles within the electrode matrix, consistent with the reference pattern reported in JCPDS card No. 05-0667.^62^ Furthermore, additional diffraction peaks located at 35.45° and 57.9° correspond to the (−111) and (202) planes of the monoclinic phase of copper(ii) oxide (CuO), in agreement with standard data;^63^; [JCPDS card No. 45-0937]. The peaks at 44.73° and 52.77° were associated with the (111) and (200) reflections of metallic copper (Cu), confirming the presence of residual metallic species;^64^ [JCPDS card No. 04-0836]. These results confirm the successful electrochemical deposition of CuO–Cu_2_O dendrites on the electrode surface, as well as the coexistence of metallic copper. Similar results were reported by Sawant *et al.*, who demonstrated the wet chemical synthesis of Cu_2_O nanoparticles and highlighted their structural and phase characteristics.^65^ Jeong *et al.* also showed that designing Cu_2_O/CuO heterostructures can significantly improve their photoelectrochemical performance.^63^

**Fig. 4:**
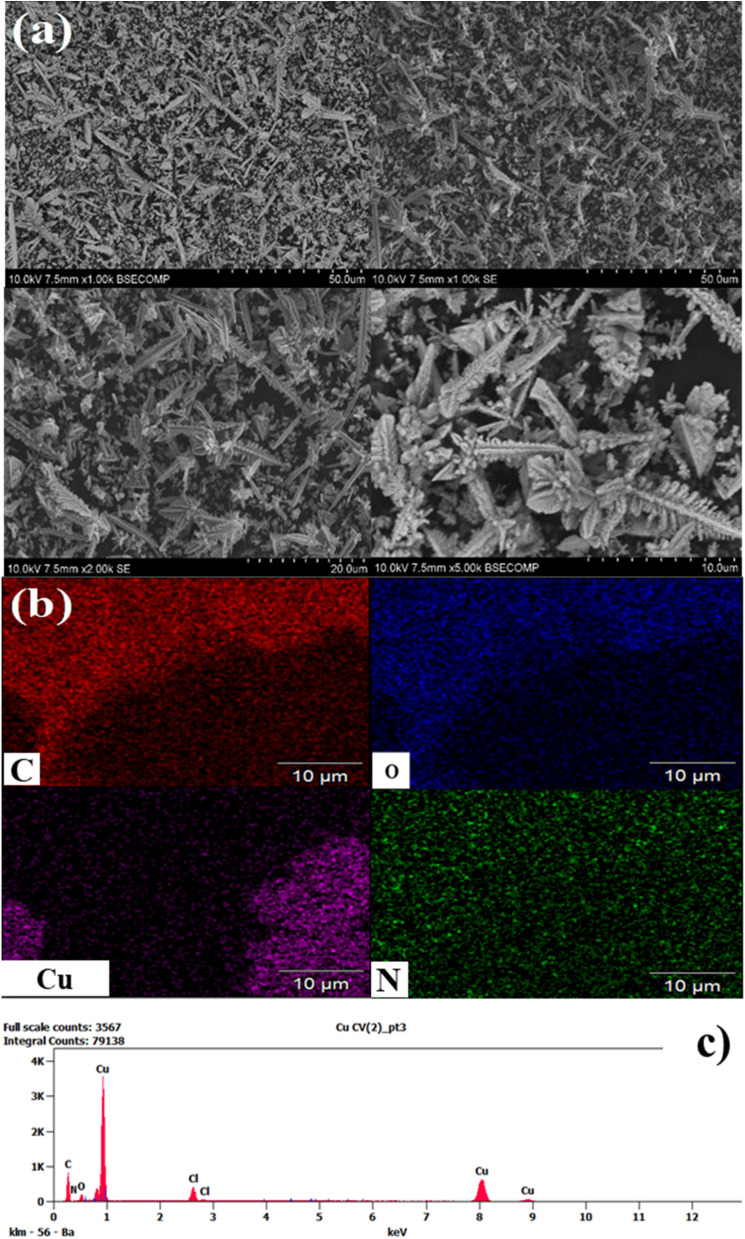
Scanning electron microscopy images of: (a) Cu Ds/poly1,5-DAN/CB/SPE, (b) elemental mapping, and (c) EDX of Cu Ds/poly 1,5-DAN/CB/SPE.

Scanning electron microscopy (SEM) together with EDX analysis was used to characterize the Cu Ds/poly 1,5-DAN film/CB (Fig. 4a and b), revealing a uniform deposition of copper across the polymer-carbon black surface, where the metal assembles into dendrites.^66,67^ This finding aligns with Zhuo *et al.*,^68^ who investigated how electrolyte composition influences the morphology of dendritic copper and showed that the presence of H_2_SO_4_ facilitates dendritic growth by enhancing nucleation and promoting anisotropic deposition. Fig. S3 (SI) presents the size distribution of copper dendrites (*n* = 100 particles), fitted to a normal distribution (mean ≈ 5.28 µm, *σ* ≈ 2.60 µm). This quantitative analysis provides statistical validation of the dendritic morphology, complementing the SEM images with numerical evidence. In addition, surface roughness was evaluated electrochemically *via* double-layer capacitance measurements, allowing estimation of the electrochemically active surface area (ECSA) and roughness factor. Together, these metrics confirm that the dendritic Cu film offers a significantly enlarged active area, directly correlating with the enhanced nitrate reduction performance.

The EDX spectrum (Fig. 4c) exhibits characteristic signals corresponding to carbon, oxygen, nitrogen, and copper, confirming the elemental composition of the modified electrode. Nitrogen is uniformly distributed over the surface, indicating the formation of a thin poly 1,5-DAN coating on the CB/SPE surface, while the presence of oxygen and copper confirms the generation of copper oxide species within the modified electrode. Previous studies have demonstrated the synergy between carbon nanostructure/polymer and copper nanoparticles; for instance, it was reported that the incorporation of functionalized MWCNTs into Cu(OH)_2_–Cu_2_O/polypyrrole composites markedly improved catalytic activity for ethanol oxidation. Similarly, Halim *et al.* synthesized hierarchical Cu_2_O–Cu(OH)_2_ nanodendrites supported on carbon nanofibers/poly(*para*-phenylenediamine), observing significantly enhanced performance for methanol electrooxidation.^69^

**Fig. 5:**
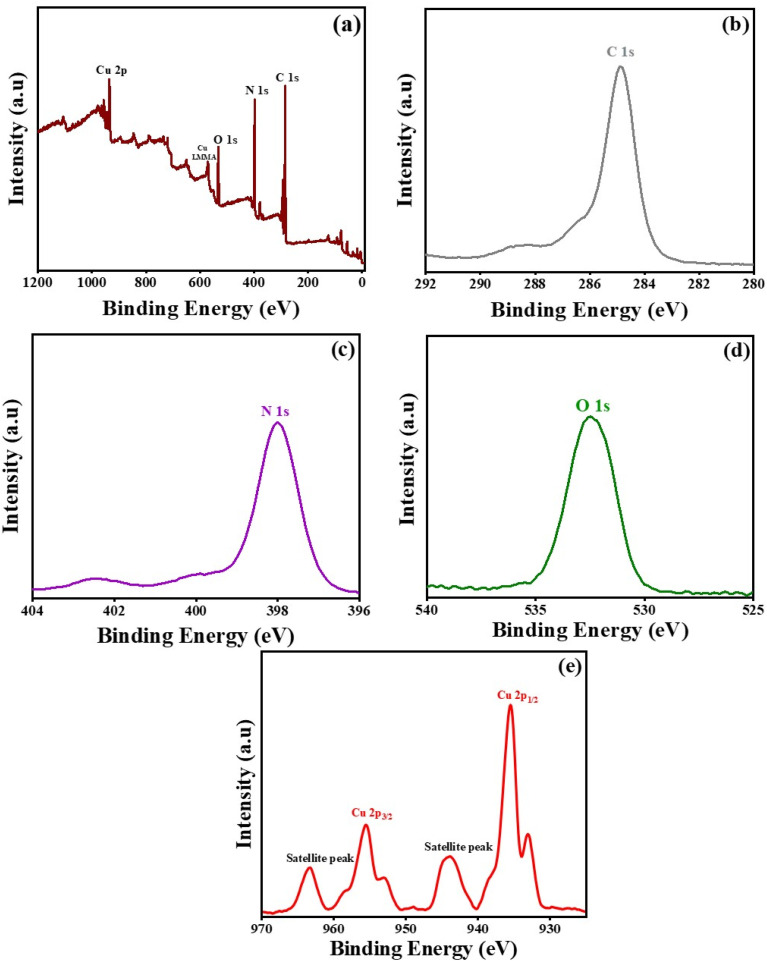
XPS analysis of Cu Ds/poly 1,5-DAN/CB/SPE: (a) survey spectrum, and high-resolution binding energy spectra of (b) C 1s, (c) N 1s, (d) O 1s, and (e) Cu 2p.

Further characterization of the Cu Ds/poly 1,5-DAN/CB/SPE electrode was performed using XPS to investigate its surface chemical composition and the oxidation states of copper present within the modified electrode. The elemental analysis revealed the presence of C (55.48%), N (1.80%), O (24.76%), Cu LMMA (1.85%), and Cu 2p (16.11%). High-resolution spectra for C, N, O, Cu LMMA, and Cu 2p are presented in Fig. 5. The C 1s spectrum displayed the main components at 284.74 eV.^70^ The N 1s spectrum displayed a peaks at 399.5 eV (C–NH_2_), confirming the presence of nitrogen-containing functional groups from the polymer matrix.^69^ The Cu LMMA spectrum exhibits a characteristic peak at 570.1 eV, which is indicative of the presence of Cu_2_O.^71^ Meanwhile, the Cu 2p high-resolution spectrum displayed two prominent peaks at 935.50 eV (42.41%) and 955.52 eV (26.29%), along with satellite peaks at 943.95 eV (16.94%) and 963.40 eV (14.37%), consistent with the Cu^2+^ oxidation state and characteristic of CuO, in addition to the metallic copper peak at 933.1 eV.^72,73,74^ The O 1s spectrum showed a major peaks at 530.43 eV (62.1%), corresponding to metal–oxygen (M–O) bonds.^72,75^ Collectively, these XPS results confirm the successful formation of a mixed-valence copper composite (CuO–Cu_2_O) on the polymer-modified electrode, with well-defined Cu(i)/Cu(ii) oxidation states and expected nitrogen- and oxygen-based functionalities.

### 3.4 Electrochemical analysis of the modified electrodes

**Fig. 6:**
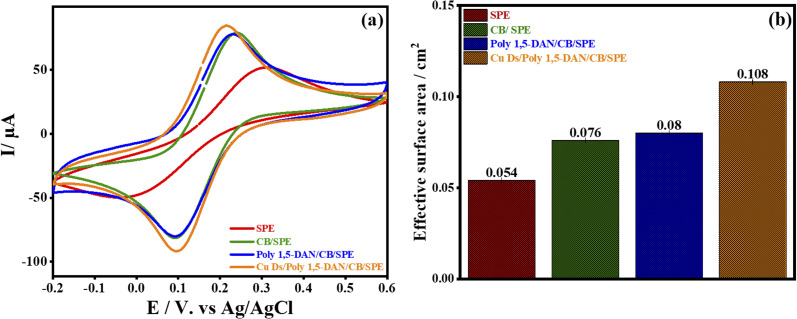
(a) Cyclic voltammograms of the bare SPE and modified SPE electrodes recorded in 5.0 mM [Fe(CN)_6_]^3−/4−^ containing 0.1 M KCl at a scan rate of 50 mV s^−1^. (b) Corresponding effective electroactive surface areas calculated from cyclic voltammograms obtained at different scan rates for the same set of electrodes.

The redox behavior of the modified electrodes was evaluated using cyclic voltammetry in a solution containing 5.0 mM [Fe(CN)_6_]^3−/4−^ and 0.1 M KCl. The resulting cyclic voltammograms for the bare SPE, CB/SPE, poly 1,5-DAN/CB/SPE and Cu Ds/poly 1,5-DAN/CB/SPE are shown in Fig. 6a. A comparative summary of the anodic and cathodic peak potentials and corresponding peak currents for each electrode configuration is provided in Table 1.

**Table 1:** Electrochemical characteristics of the anodic and cathodic peaks for the bare and modified SPEs

Electrode	*I*_pa_/µA	*I*_pc_/µA	*E*_pa_/mV	*E*_pc_/mV	Δ*E*_p_/mV
SPE	51	−49	295	−20	315
CB/SPE	77	−79	237	91	146
Poly 1,5-DAN/CB/SPE	78	−80	228	93	135
Cu Ds/poly 1,5-DAN/CB/SPE	87	−92	220	93	127

For the bare SPE, the redox couple exhibited a Δ*E*_p_ of roughly 315 mV, indicative of relatively slow electron-transfer kinetics at the interface. A significant enhancement in current response was observed after the incorporation of carbon black (CB) onto the surface of the SPE (green curve). The modified electrode exhibited an anodic peak current of approximately 78 µA with a peak-to-peak separation of about 146 mV, compared to the 51 µA observed for the bare SPE (red curve). This enhancement is attributed to the superior electrical conductivity and high surface area of carbon black nanoparticles, as widely reported in the literature.^76^ Upon modification with a poly 1,5-DAN film, the recorded current decreased slightly to approximately 76 µA. This slight reduction can be attributed to the decrease in conductivity of the polymer under neutral pH conditions. However, we can consider that in the presence of CB, the response of the poly 1,5-DAN is improved even at neutral pH. Interestingly, despite the drop in current, a marked decrease in the peak-to-peak separation was observed for the poly 1,5-DAN/CB/SPE (blue curve), reaching 135 mV. This narrowing of Δ*E*_p_ suggests that poly 1,5-DAN exhibits electrocatalytic activity, thereby facilitating electron transfer at the interface. A comparable trend was reported by Halim *et al.*, who observed that introducing a diamine-based polymer within a carbon nanostructured matrix can significantly enhance interfacial charge transfer, even when the polymer itself is not highly conductive under neutral conditions. In their system, the presence of poly(*para*-phenylenediamine) improved the electron-transfer kinetics at the Cu_2_O–Cu(OH)_2_/CNF interface, leading to a noticeable reduction in Δ*E*_p_ and a more favorable electrochemical response. Likewise, Shi *et al.* demonstrated that poly(1,5-diaminonaphthalene) deposited on carbon nanotubes facilitated electron transfer during nitrite oxidation, despite a modest decrease in the ferro/ferricyanide current.^77,78^

Following the electrochemical deposition of copper oxide dendrites onto the poly 1,5-DAN/CB layer, the peak current further increased to 87 µA (orange curve). This enhancement confirms the successful formation of copper-based particles on the poly 1,5-DAN/CB surface, which is in agreement with the observations of Halim *et al.*^77^ This outcome can be attributed to the synergistic interaction between carbon-based materials, polymers, and copper particles, which effectively increases the available surface area and facilitates electron transfer at the electrode interface, as reported by several researchers.^24,79^

To determine the electrochemically active surface area of the electrodes, cyclic voltammetry was performed at scan rates ranging from 10 to 200 mV s^−1^ in a 5.0 mM [Fe(CN)_6_]^3−/4−^ solution prepared in 0.1 M KCl. The effective surface area was then calculated using the Randles–Ševčík eqn (1):^80,81^

1 *I*_p_ = 2.69 × 10^5^ × *n*^3/2^ × *C* × *A* × *D*^1/2^ × *v*^1/2^

In eqn (1), *v* denotes the scan rate (V s^−1^), *n* is the number of electrons involved in the redox process (*n* = 1), *C* is the concentration of [Fe(CN)_6_]^3−/4−^ (5 × 10^−6^ mol cm^−3^), *D* is the diffusion coefficient of the redox probe (7.6 × 10^−6^ cm^2^ s^−1^), and *A* represents the electroactive surface area (cm^2^). *I*_p_ corresponds to the peak current (A).

**Fig. 7:**
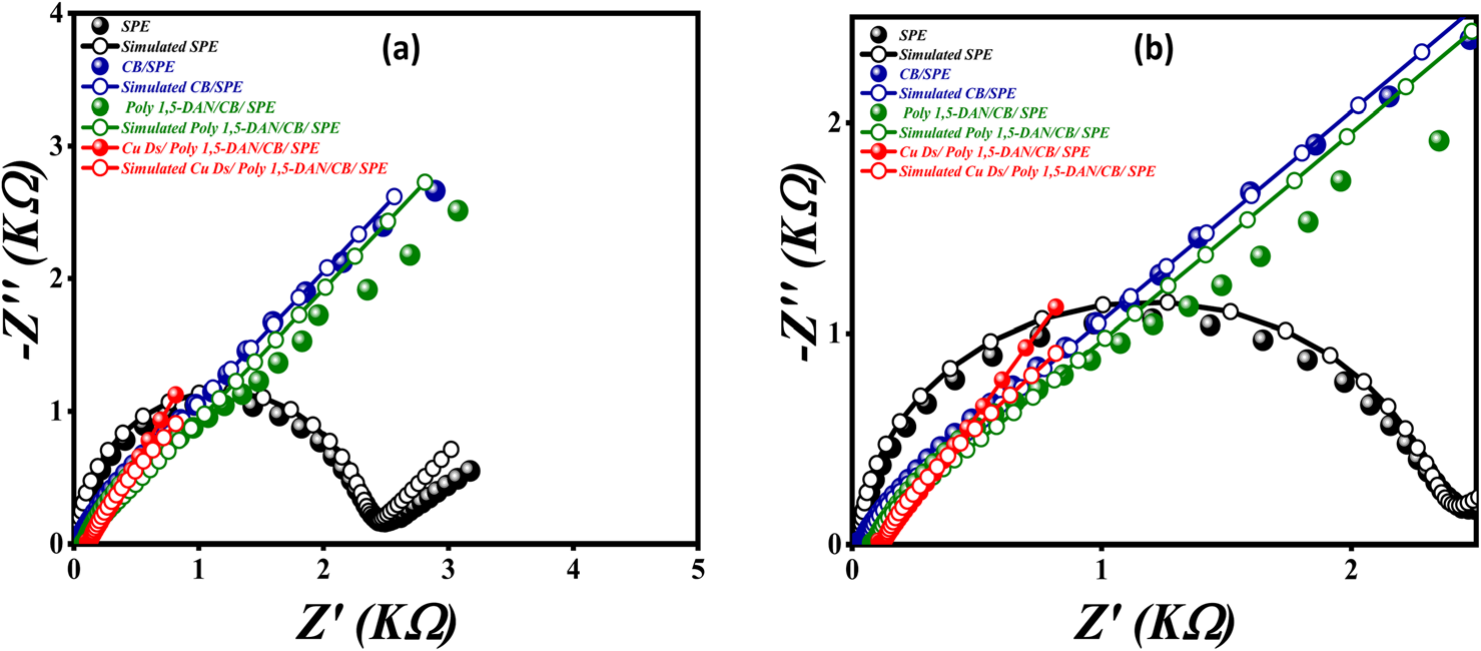
(a) Nyquist plots of SPE, CB/SPE, poly 1,5-DAN/CB/SPE and Cu Ds/poly 1,5-DAN/CB/SPE, recorded in 5.0 mM [Fe(CN)_6_]^3−/4−^ containing 0.1 M KCl. (b) Zoomed-in view of the 0–2600 Ω region.

The Cu Ds/poly 1,5-DAN/CB/SPE exhibited a pronounced enhancement, indicating a substantial increase in electroactive surface area (0.1079 cm^2^) in comparison with the unmodified SPE (0.0539 cm^2^) as shown in Fig. 6b. This increase is consistent with the improved electrochemical response toward Fe(CN)_6_^3−/4−^ ions observed in cyclic voltammetry, where copper particles act as active catalytic sites, facilitating ferrocyanide reduction and expanding the effective surface area. Such enhancement directly contributes to greater sensitivity and efficiency in the detection process. Moreover, the synergistic integration of copper with poly 1,5-DAN and carbon black further amplified the electroactive surface area compared to CB/SPE (0.076 cm^2^) and poly 1,5-DAN/CB/SPE (0.08 cm^2^), highlighting the synergy of the polymer matrix and carbon support in dispersing copper particles and promoting efficient electron transfer. Electrochemical impedance spectroscopy was employed to investigate the interfacial charge – transfer properties of the bare and modified SPEs. The Nyquist plots for the SPE, CB/SPE, poly 1,5-DAN/CB/SPE, and Cu Ds/poly 1,5-DAN/CB/SPE, recorded in a 5.0 mM [Fe(CN)_6_]^3−/4−^ solution containing 0.1 M KCl over a frequency range of 100 kHz to 10 mHz, are shown in Fig. 7a. The charge-transfer resistance *R*_ct_ and film capacitance (*C*_f_) values were obtained by fitting the impedance spectra using the Randles equivalent circuit. A summary of the extracted parameters is presented in Table 2.

**Table 2:** Impedance characteristics obtained through fitting of the EIS data

Electrode	*R*_ct_ (Ω)	*C*_f_ (µF)
SPE	2280 ± 50	0.507
CB/SPE	35 ± 1.5	176.7
Poly 1,5-DAN/CB/SPE	41 ± 1.85	135.7
Cu Ds/poly 1,5-DAN/CB/SPE	2.65 ± 0.2	204

For the bare SPE, the Nyquist plot reveals a prominent semicircle at high frequencies region, which corresponds to a high charge transfer resistance (*R*_ct_ ≈ 2280 Ω). In contrast, the linear segment observed at lower frequencies indicates a diffusion-controlled process occurring at the electrode interface.^77^ After incorporating CB onto the SPE surface, the charge transfer resistance significantly decreased to approximately 35 Ω. This improvement can be attributed to the high electrical conductivity and large specific surface area of CB, which facilitates rapid electron transfer and enhance charge transport at the electrode-solution interface. Following the deposition of the poly 1,5-DAN film on the modified electrode, further changes in the interfacial electrochemical behavior were observed. The charge transfer resistance decreased markedly to 41 Ω in comparison with bare SPE, indicating enhanced conductivity and increased electroactive surface area of the polymer, which together promote increased electron-transfer efficiency at the sensor interface. Based on these findings, the electrochemical polymerization of poly 1,5-DAN onto the CB film generates a synergistic effect that significantly enhances the electrical properties of the resulting composite, thereby facilitating more efficient electron transfer at the electrode interface. The electrodeposition of Copper onto the poly 1,5-DAN/CB modified surface led to a further reduction in the charge-transfer resistance to 2.65 Ω. This improvement can be attributed to the well distribution of copper species within the poly 1,5-DAN/CB matrix, which generate a high density of electroactive sites and promotes electron-transfer kinetics. The EIS results are fully consistent with the CV findings and corroborate previous studies, thereby confirming the superior electrochemical properties of the resulting hybrid electrodes.^24,77,82^

### 3.5 Electrochemical detection performance of the sensor toward NO_3_^−^

**Fig. 8:**
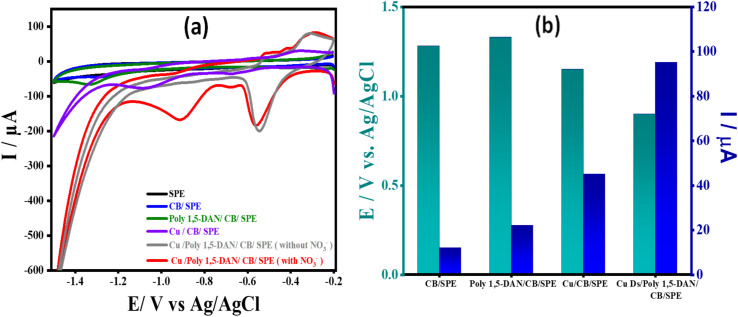
(a) CV of SPE, CB/SPE, poly1,5-DAN/CB/SPE, Cu/CB/SPE, Cu Ds/poly 1,5-DAN/CB/SPE in the presence and the absence of 1.0 mM NO_3_^−^ in 0.1 M Na_2_SO_4_ (pH = 5) at 50 mV s^−1^. (b) Variation of NO_3_^−^ peak currents and potentials *vs.* different WEs.

To evaluate the sensing capability of the prepared electrode, the electrochemical response of the Cu Ds/poly 1,5-DAN/CB/SPE towards nitrate reduction was evaluated by cyclic voltammetry. Measurements were performed in a solution containing 0.1 M Na_2_SO_4_ and 1.0 mM of NO_3_^−^ over the potential range of −0.2 V to −1.5 V *versus* the Ag/AgCl reference electrode. The resulting voltammograms, shown in Fig. 8a, clearly demonstrate the reduction process of nitrate ions occurring at the surface of the modified electrodes.

As anticipated, the bare SPE exhibited a very weak response of −1 µA, confirming that nitrate ions undergo negligible reduction on its surface. Upon drop-casting carbon black onto the SPE, the current response increased to −12 µA. Further modification of the CB/SPE with poly 1,5-DAN slightly enhanced the electrochemical signal towards nitrate reduction, yielding a current of −22 µA. In contrast, when copper particles were incorporated onto the CB/SPE surface, a peak at −1.15 V *vs.* Ag/AgCl was observed, attributed to the reduction of nitrate ions, with a significantly higher current of – 45 µA. This pronounced enhancement can be attributed to copper's catalytic activity, which accelerates electron transfer and promotes nitrate reduction. Indeed, previous studies have demonstrated that copper effectively facilitates the electroreduction of NO_3_^−^ under both acidic and neutral conditions.^37,83^

The electrode modified with copper particles and poly 1,5-DAN/CB exhibited a markedly enhanced electrochemical response toward nitrate ions (NO_3_^−^). As shown in Fig. 8a, two distinct peaks appeared: the first, at −0.56 V, corresponds to the reduction of Cu(ii) (eqn (2)), The second peak observed at −0.92 V is attributed to the electrochemical reduction of NO_3_^−^, producing a current of −95 µA. This current is approximately twice the magnitude obtained with the Cu Ds/CB/SPE system and is accompanied by a potential shift of about 230 mV (Fig. 8b). A comparable behavior has also been reported in earlier work by Motaghedifard *et al.*^82^ The deposition of the poly 1,5-DAN layer on the CB/SPE surface helps inhibit the agglomeration of copper oxide particles, promoting a more uniform distribution and improved stability of the catalytic material,^84^ thereby contributing to improved dispersion. In addition, a copper-related peak appears at −0.56 V, which may be attributed to the formation of a complex involving copper, the polymer matrix, and carbon black. The enhanced nitrate-reduction response is primarily associated with the uniform distribution of copper particles, which facilitates efficient electron transfer. Copper is well known for its catalytic ability to accelerate electron-transfer processes, thereby improving the overall electrochemical performance of the sensor, as well as its selectivity in the reduction and detection of NO_3_^−^, as highlighted in previous studies.^38,85^ The observed enhancement in current density for the Cu Ds/poly 1,5-DAN/CB/SPE system can thus be attributed to the synergistic interaction between the composite structure and the poly 1,5-DAN film. The polymer matrix not only promotes the homogeneous dispersion of copper oxide across the electrode surface but also enhances charge-transfer pathways by providing a conductive and electroactive environment that complements the catalytic activity of the copper species but also provides amine functional groups that interact with both carbon black and copper, thereby enhancing the electrode's sensitivity toward nitrate detection. The remarkable electrocatalytic performance of the Cu Ds/poly 1,5-DAN/CB/SPE electrode toward nitrate reduction originates from the strong interfacial synergy between the conductive carbon black scaffold, the nitrogen-rich poly 1,5-DAN matrix, and the multivalent copper active sites (Cu^0^/Cu^+^/Cu^2+^). Electropolymerized poly 1,5-DAN plays a dual structural and electronic role by providing a high density of amine coordination sites capable of chelating copper species while simultaneously stabilizing the dendritic Cu architecture against aggregation and surface oxidation.^24,29^ This coordination environment modulates the electronic density of the Cu centers, promotes the formation of highly dispersed small Cu active sites, and enhances the adsorption and activation of nitrate-derived intermediates. Quantitatively, this effect results in a higher density of electrochemically accessible catalytic sites compared with the unmodified electrodes. In parallel, carbon black significantly increases the electrochemically active surface area (ECSA), improves electrical conductivity, decreases interfacial charge-transfer resistance, and facilitates rapid electron transport across the electrode/electrolyte interface, thereby enabling higher reduction currents at lower overpotentials and ensuring homogeneous current distribution throughout the catalytic layer, as reported by Chen *et al.*^86^ Halim *et al.* demonstrated a comparable trend in their study on hierarchical Cu_2_O–Cu(OH)_2_ nanodendrites supported on carbon nanofibers/poly(*para*-phenylenediamine) nanocomposites, emphasizing the critical role of polymeric supports and carbon-based frameworks in enhancing electrocatalytic activity.^77^ Similarly, Wangchuk *et al.* reported the development of cuprous oxide-functionalized activated porous carbon-modified screen-printed carbon electrodes integrated with a smartphone platform for portable electrochemical nitrate detection, underscoring the importance of copper in such sensing applications.^87^ In another related work, Vu *et al.* investigated copper ion interactions with poly(1,8-diaminonaphthalene)/graphene films for the voltametric determination of pyridoxine, further highlighting the versatility of this polymer in electrochemical systems.^88^ Collectively, these findings reinforce a significant contribution of polymeric and carbon-based materials, when combined with copper species, in contributing to improved performance of electrochemical sensing and catalytic applications.

Based on the electrochemical behavior observed in this work and findings from the literature,^42,89^ the following reactions can be proposed for nitrate reduction. Under sufficiently negative potentials, aqueous NO_3_^−^(aq) is first adsorbed onto the electrode surface, forming NO_3_^−^(ads), which was then reduced to NO_2_^−^(ads) (3 & 4) and finally to NH_4_^+^ (5).^5,90,91^ Within this mechanism, the poly 1,5-DAN layer stabilizes the reaction intermediates through interfacial interactions and tunes their adsorption geometries, while the carbon black network ensures efficient electron percolation and rapid charge propagation. Simultaneously, the homogeneous dispersion of Cu dendrites increases the density of equivalent active sites and lowers the activation barriers associated with hydrogenation steps. The superior performance of the Cu Ds/poly 1,5-DAN/CB/SPE electrode arises from the combined contribution of copper dendrites, poly 1,5-DAN and carbon black. This synergistic architecture enhances nitrate reduction, leading to higher currents, improved selectivity, better stability, and excellent reproducibility compared with CB/SPE and poly 1,5-DAN/CB/SPE electrodes.

2 Cu^2+^ + 2e^−^ → Cu

3 NO_3(aq)_^−^ → NO_3(ads)_^−^

4 NO_3(ads)_^−^ + 2H^+^ + 2e^−^ → NO_2(ads)_^−^ + H_2_O

5 NO_2_^−^ + 8H^+^ + 6e^−^ → NH_4_^+^ + 2H_2_O

6 NO_3_^−^ + 10H^+^ + 8e^−^ → NH_4_^+^ + 3H_2_O

### 3.6 Optimization of electrochemical parameters

#### 3.6.1 Effect of the electropolymerization mode

For comparative analysis, the electrochemical response of NO_3_^−^ was investigated on electrodes prepared using both cyclic voltammetry (CV) and galvanostatic mode. Measurements were carried out in an aqueous medium of 0.1 M Na_2_SO_4_ with 1.0 mM NO_3_^−^. As shown in Fig. S4 (SI), the electrode fabricated *via* CV mode exhibited a superior current response compared to that obtained by the galvanostatic method. Based on these findings, subsequent poly 1,5-DAN preparation experiments were carried out using the CV approach.

#### 3.6.2 Optimization of the number of CV cycles used for copper electrodeposition

The amplitude of the NO_3_^−^ reduction peak is affected by the processing parameters of copper electrodeposition, including the scan rate, deposition time, and the number of deposition cycles performed by cyclic voltammetry, as previously demonstrated by Li *et al.*^92^ To achieve optimal deposition performance, four different CV cycle numbers (2, 5, 10, and 15) were applied to fabricate Cu Ds/poly 1,5-DAN/CB/SPE electrodes in a solution containing 0.1 M H_2_SO_4_ and 0.1 M CuSO_4_. Following electrodeposition of copper, each electrode was evaluated in 0.1 M Na_2_SO_4_ containing 1.0 mM NO_3_^−^. As shown in Fig. S5a (SI), the reduction peak current varied with the number of deposition cycles, with the electrode prepared using ten CV cycles exhibiting the highest current response. This result indicates that an optimal number of deposition cycles is required to achieve maximum catalytic activity, as insufficient cycles lead to incomplete copper coverage, while excessive cycling may cause particle agglomeration or film thickness, thereby reducing electroactive surface accessibility.

The thickness of the electropolymerized poly 1,5-DAN layer and copper film was estimated from the integrated deposition charge using Faraday's law (eqn (7)).7
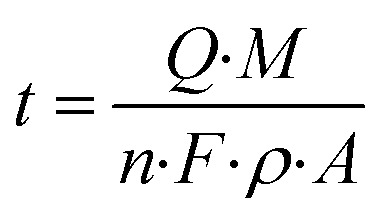
*t* represents the film thickness, *Q* is the total charge passed during deposition (C), *M* is the molar mass of the deposited material (g mol^−1^), *n* is the number of electrons involved in the reduction reaction, *F* is the faraday constant (96 485 C mol^−1^), *ρ* is the density of the copper (8.96 g cm^−3^), and *A* is the electrode surface area (cm^2^), was approximately 0.07 µm. The Cu film thickness was calculated to be ≈1.97 µm for 10 deposition cycles (*Q* = 0.265 C), while values of 0.395, 0.985, and 2.94 µm were obtained for 2, 5, and 15 cycles as shown in Table 3, respectively (Fig. S5b, SI). Increasing the Cu film thickness enhanced the electrochemically active surface area and promoted the formation of defect-rich dendritic structures favorable for nitrate reduction. However, excessively thick Cu layers negatively affected mass transport and electron-transfer kinetics, resulting in lower catalytic efficiency. These findings demonstrate that controlling the Cu film thickness is essential to balance active-site density, conductivity, and nitrate diffusion, thereby maximizing the sensing performance.^92^ This result is in agreement with to the previous work of Halim *et al.*^77^

**Table 3:** Effect of CV cycle number on nitrate response and Cu film thickness

CV cycle number	Response of nitrate ions (µM)	Cu film thickness (µm)
2	−63	0.395
5	−74	0.985
10	−95	1.97
15	−82	2.94

#### 3.6.3 Electrochemical kinetics of nitrate reduction

**Fig. 9:**
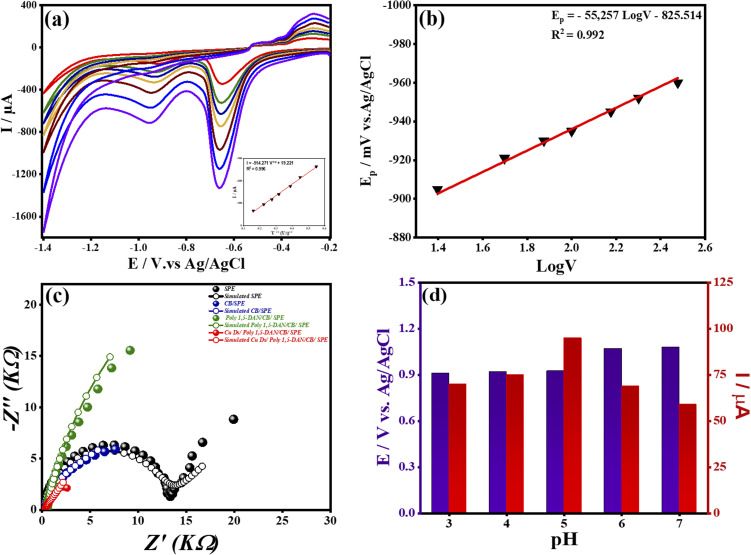
(a) Cyclic voltammograms of the Cu-Ds/poly 1,5-DAN/CB/SPE recorded in 0.1 M Na_2_SO_4_ containing 1.0 mM NO_3_^−^ at scan rates from 25 to 300 mV s^−1^, along with the corresponding plot of the nitrate-reduction peak current *versus* V^1/2^, (b) relationship between the peak potential (*E*_p_) of the nitrate-reduction peak and log(*V*), (c) Nyquist plots for SPE (black), CB/SPE (blue), poly 1,5-DAN/CB/SPE (green), and Cu-Ds/poly 1,5-DAN/CB/SPE (red) recorded in 0.1 M Na_2_SO_4_ containing 1.0 mM NO_3_^−^. (d) Variation of the nitrate-reduction peak current and peak potential as a function of pH.

To better understand the electrode kinetics, the effect of scan rate on nitrate reduction was assessed in the same electrolyte (Fig. 9a) over 25–300 mV s^−1^. The resulting plot of *E*_p_*versus* V^1/2^ exhibited a clear linear trend, which can be expressed as: *I*_p_ = −514.271 V^1/2^ + 19.221, and (*R*^2^ = 0.996). These findings imply that nitrate reduction at the modified electrode is governed by a diffusion-controlled process.^93^ A comparable behavior was reported by Anurag *et al.* when employing a graphene oxide-MWCNT composite with electrochemically deposited copper nanoparticles on screen-printed electrodes, and by Inam *et al.* when using a flexible screen-printed electrochemical sensor functionalized with electrodeposited copper.^29,46^ Furthermore, the linear relationship obtained from the plot of *E*_p_*versus* log *V* confirms the irreversible nature of the electrode process: (*E*_pc_ = – 55.257 log(*V*) − 825.514, *R*^2^ = 0.992) as shown in Fig. 9b.

To further investigate the electrode kinetics, the half-peak potential (*E*_p/2_) was analyzed.^46^ The magnitude of (Δ*E*_p/2_ = *E*_p_ − *E*_p/2_) was calculated and plotted as a function of scan rate (Fig. S6, SI). Results revealed that Δ*E*_p/2_ remained essentially constant across the scan rate range of 25–300 mV s^−1^, yielding an average value of 70 ± 7 mV. The stability of this parameter indicates that the charge transfer coefficient involved in the electrochemical reduction of NO_3_^−^ does not vary with the scan rate, confirming the stability of the electron-transfer kinetics under the tested conditions.

Understanding the electrochemical reduction mechanism relies heavily on the influence of the scan rate. To determine the number of electrons participating in the reaction, the relationship between the peak current and the scan rate is typically examined. In this work, the peak potential exhibited a linear dependence on the square root of the scan rate, suggesting that the process is governed by diffusion-controlled kinetics. As previously discussed, nitrate reduction can proceed through multiple pathways; therefore, the number of electrons transferred was determined using eqn (8), based on Laviron's model for irreversible electrode processes.^29,93,94^8
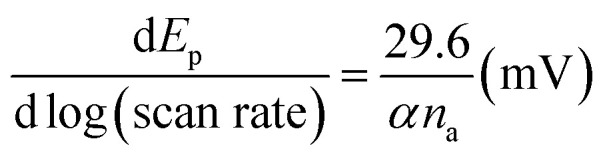


To quantify kinetic parameters, the transfer coefficient *α*, the number of electrons in the rate-determining step *n*_a_, and the peak potential *E*_p_ were related *via* Laviron's expression. Based on the inset-derived slope of Fig. 9b, *αn*_a_ was determined to be 0.535. The number of e^−^ involved was then estimated from the dependence of the peak current on the scan rate, as expected for an irreversible electrochemical process. As the scan rate increases, the diffusion layer thickness decreases, leading to higher peak currents. The influence of scan rate on nitrate reduction at the sensor was examined (Fig. 9a), and the data were consistent with a totally irreversible, diffusion-controlled process, described by:^42,95,96^9
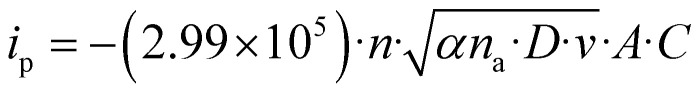


In this equation, *A* represents the active surface area of the electrode, *C* is the bulk concentration of nitrate (mol cm^−3^), *n* denotes the number of electrons transferred during the reaction, *α* is the charge transfer coefficient, *D* corresponds to the diffusion coefficient of nitrate (2.0 × 10^−5^ cm^2^ s^−1^), and *ν* is the scan rate, which in this study was varied between 25 and 300 mV s^−1^ (Fig. 9a).^20^

The Cu Ds/poly 1,5-DAN/CB/SPE exhibited pronounced nitrate reduction activity, with the reduction current found to be directly proportional to the nitrate concentration or This behavior indicates that the reduction process is governed primarily by the diffusion rate of nitrate ions toward the electrode rather than by intrinsic electrode properties. The number of heterogeneous electrons transferred (*n*) was estimated from eqn (9), resulting in a value close to 8 (*n* = 8.33), which is consistent with the overall nitrate reduction pathway described in eqn (6). As supported by previous studies, the electrochemical reduction of nitrate typically proceeds through an initial adsorption of nitrate onto the electrode surface, followed by its stepwise reduction to nitrite and subsequently to ammonium NH_4_^+^, as outlined in eqn (4) and (5).^47,92,97^ The incorporation of copper dendrites, Poly 1,5-DAN, and CB into the screen-printed electrode significantly enhances this process, providing abundant active sites and facilitating efficient electron transfer. The adsorption step plays a pivotal role in this mechanism, as it promotes stronger interaction between nitrate ions and the catalytic surface, thereby improving charge transfer efficiency and enabling more complete nitrate reduction.

Electrochemical impedance spectroscopy was performed in the same electrolyte to validate results obtained from the CV measurements. The corresponding Nyquist plots are presented in Fig. 9c. A clear difference can be observed among the responses of the bare SPE (black line), CB/SPE (blue line), poly 1,5-DAN/CB/SPE (green line), and Cu Ds/poly 1,5-DAN/CB/SPE (red line). After electrode modification, the charge transfer resistance (*R*_ct_) decreased, dropping from 13.4 kΩ for the unmodified SPE to 0.163 kΩ for the Cu Ds/poly 1,5-DAN/CB/SPE. This substantial reduction indicates improved interfacial electron transfer and suggests that nitrate ions can diffuse more efficiently toward the electrode surface.^20^ Based on the CV and EIS results, it can be concluded that modification of the SPE significantly enhanced the electrochemical performance of the electrode toward nitrate detection.^47^

#### 3.6.4 Influence of pH

Electrolyte pH is another critical parameter that must be carefully controlled during the electrochemical process. Variations in pH not only influence the structural properties of the DAN but also directly impact the electrochemical polymerization process, stability of the deposited metal film, and, most critically, the electrochemical reduction of nitrate. Consequently, the electrolyte pH plays a decisive role in governing the electrochemical response toward NO_3_^−^.^98^ The procedure was highly dependent on the experimental environment, including the pH, the type of electrode used, and the composition of the supporting electrolyte. To investigate this effect, the electroreduction of nitrate was performed in 0.1 M Na_2_SO_4_ containing 1 mM nitrate at different pH values (Fig. 9d). The results revealed that pH values lower than 5 or higher than 5 were unfavorable for nitrate reduction, while highly acidic conditions also hindered polymer synthesis. At pH = 5, the voltammogram exhibited the highest peak current, indicating that this condition provides the most favorable environment for efficient nitrate reduction.

### 3.7 Electrochemical detection of nitrate ions NO_3_^−^

**Fig. 10:**
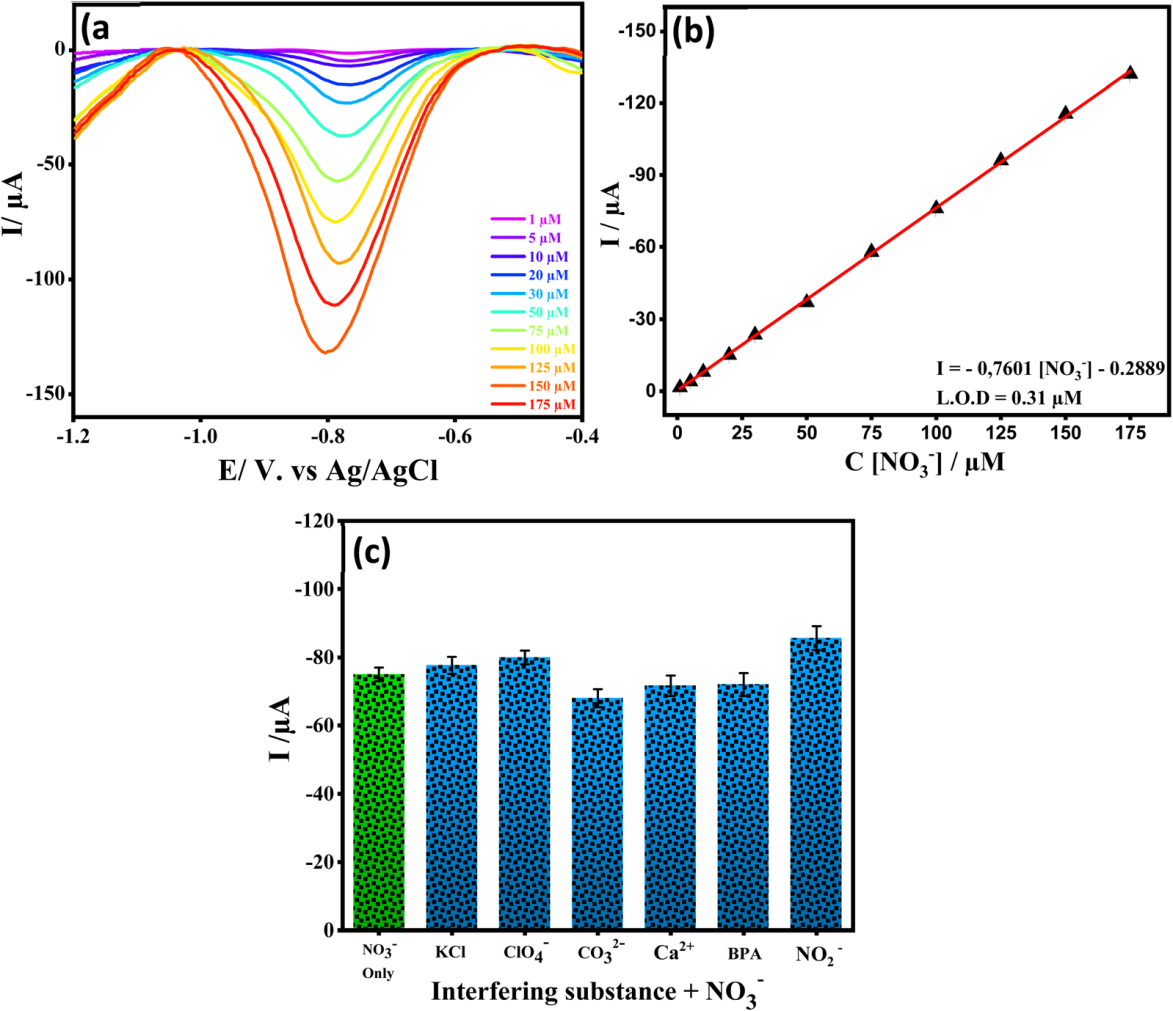
(a) SWV responses of Cu Ds/poly 1,5-DAN/CB/SPE *versus* concentrations of NO_3_^−^ (1–175 µM). (b) Corresponding calibration curve constructed from the SWV peak currents. (c) Variation in the nitrate reduction current under conditions involving typical potential interferents at fifty-fold excess concentrations, recorded using the sensor.

To assess the analytical capability of the developed sensor for nitrate detection, its electrochemical response was recorded and analyzed. The Cu Ds/poly 1,5-DAN/CB/SPE was examined under optimized conditions and recorded across a range of NO_3_^−^ concentrations. SWV was made using 0.1 M Na_2_SO_4._ The resulting voltammograms for nitrate concentration ranging from 1.0 µM to 175 µM are displayed in Fig. 10a.

As illustrated in Fig. 10b, a strong linear correlation was observed between the peak reduction current and the nitrate concentration, with an excellent correlation coefficient of 0.999. Square-wave voltammetry was employed to assess the sensor's analytical performance, including its sensitivity, linear dynamic range, and LOD of the elaborated sensor toward nitrate ion sensing. The calibration plot demonstrated A linear relationship was obtained between the reduction current and the nitrate concentration, described by the regression equation: *I*_pa_ (µA) = (−0.7601) [NO_3_^−^] (µM) − 0.2889, *R*^2^ = 0.999 (Fig. 10b). The LOD was subsequently calculated using the standard deviation method (eqn (10)), confirming the high analytical performance of the Cu Ds/poly 1,5-DAN/CB/SPE electrode.^99^

10 LOD = 3*σ*/*S*

where *σ* denotes the standard deviation of the response, typically obtained from blank concentration samples (*n* = 10), and *S* represents the slope of the calibration curve. Based on this relationship, the limit of detection (LOD) was calculated to be 0.28 µM, with a sensitivity of 7.0 µA µM^−1^ cm^−2^. The fabricated sensor exhibits an extended linear range, a low detection limit, and high sensitivity compared with recently reported electrochemical nitrate sensors, highlighting its superior analytical performance (Table 4). Consequently, the Cu Ds/poly 1,5-DAN/CB/SPE provides a reliable and efficient platform for electrochemical nitrate sensing. Moreover, unlike sensors based on expensive noble metals such as gold, silver, or platinum, the proposed material offers a cost-effective alternative while maintaining high analytical performance.

**Table 4:** Comparative analysis between this work and recent reports on electrochemical nitrate sensors

Electrode	Method	Linear range (µM)	LOD (µM)	pH	*R* ^2^	Ref.
MPCA/PGE	DPV	0.8–30.0	0.09	5	0.9991	82
Cu Ds/ERGO-MWCNT/SPCE	SWV	10–750	3.3	2	0.994	29
Cu2O@APC/SPE	Chronoamperometry	4.0–1000	1.2	2.7	0.9983	87
CdS nanorods/GSPE	CV	50–5000	2.3	8	0.999	100
Graphene-Cu/Si	DPV	10–90	7.89	2	—	101
Cu modified carbon SPE	Amperometry	100–20000	—	7	0.9972	102
Cu/SPCE	LSV	0.05–3000	0.87	7	0.9928	103
Cu/MWCNT/RGO/GCE	SWV	0.1–75	20 × 10^−3^	3	0.9979	104
Cu@Fe_3_O_4_/Au electrode	DPV	10–1000	1.35	2	0.9914	105
CuPc-N-MWCNTs-SPCE	DPV	50–1000	20.0	7	0.9984	106
Cu Ds/poly 1,5-DAN/CB/SPE	SWV	1–175	0.28	5	0.999	This work

Assessing the overall performance of the sensor requires careful assessment of its stability, reproducibility, and repeatability. The stability of the Cu Ds/poly 1,5-DAN/CB/SPE was assessed over one week, during which the electrode retained approximately 90% of its initial current response, indicating good operational stability for nitrate detection (Fig. S7, SI). Reproducibility was examined using a set of five independently prepared electrodes (Fig. S8, SI), yielding a relative standard deviation (RSD) of 3%, which demonstrates the outstanding fabrication uniformity. Furthermore, the repeatability was exhibited for the detection of 100 µM NO_3_^−^. After five successive measurements with the same sensor, the RSD was calculated to be 3.39% (Fig. S9, SI), confirming the sensor's reliable and repeatable performance.

### 3.8 Study of interferences

The selectivity of the Cu Ds/poly 1,5-DAN/CB/SPE sensor toward NO_3_^−^ sensing was also evaluated using SWV in the presence of potentially interfering species (KCl, CO_3_^2−^, ClO_4_^−^, Ca^2+^, BPA, and NO_2_^−^) as shown in Fig. 10c, each added at a fifty-fold excess relative to nitrate ions. The peak current for nitrate in well water was −75 µA, while the successive addition of the interferents produced peak currents of −77.67, −80, −68, −71.67, −72, and −85.67 µA, corresponding to variations of +2.67 µA (∼3.6%), +5 µA (∼6.7%), −7 µA (∼9.3%), −3.33 µA (∼4.4%), −3 µA (∼4%), and −10.67 µA (∼14.2%), respectively. The small changes observed for most interferents indicate negligible interference, with only NO_2_^−^ causing a slightly larger decrease, likely due to its participation in similar electrochemical processes. Overall, these results confirm that the Cu Ds/poly 1,5-DAN/CB/SPE sensor maintains outstanding selectivity for nitrate sensing, even in the presence of high concentrations of common coexisting ions.

### 3.9 Nitrate measurement in real water matrices

**Fig. 11:**
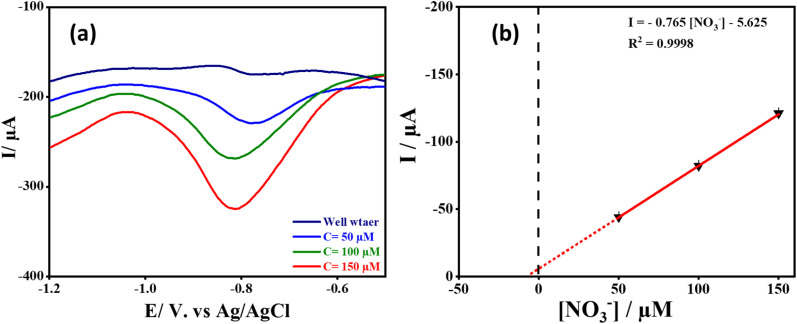
(a) SWV curve for nitrate detection in well water under optimized experimental conditions using Cu Ds/poly 1,5-DAN/CB/SPE. (b) Corresponding standard addition curve.

To validate the analytical applicability of the developed sensor for nitrate detection in real water samples, the standard additions method was employed by spiking well water with 50 µM, 100 µM, and 150 µM of NO_3_^−^ (Fig. 11a). SWV was performed under optimized experimental conditions,^107^ yielding well-defined peaks at −0.83 V *vs.* Ag/AgCl. The corresponding peak currents showed a clear correlation with the added nitrate concentrations (Fig. 11b and Table 5). As shown in Table 5, the recovery and precision results demonstrate the reliability of the proposed sensor. For a spiked concentration of 50 µM, the sensor detected 52.5 ± 0.89 µM, corresponding to a recovery of 105% with an RSD of 1.7%. At 100 µM, the measured value was 104.5 ± 2.92 µM, yielding a recovery of 104.5% and an RSD of 2.8%. Finally, for 150 µM, the sensor response was 155 ± 4.96 µM, giving a recovery of 103.3% with an RSD of 3.2%. These results confirm both the accuracy and reproducibility of the method, with recoveries consistently above 100% and low RSD values, underscoring the suitability of the sensor for practical nitrate determination in real samples. The calculated recoveries for the well water samples were close to 100%, with relative standard deviations (RSDs) below 3.2%, confirming the sensor's high accuracy, precision, and reliability for monitoring NO_3_^−^ in real water samples.

**Table 5:** Recovery and relative standard deviation (RSD) values for nitrate determination in well water samples using the elaborated sensor (*n* = 3)

Sample	Added (µM)	Found (µM)	Recovery (%)	RSD (%) (*n* = 3)
Well water	50	52.5	105	1.7
	100	104.5	104.5	2.8
	150	155	103.33	3.2

## 4 Conclusion

In this work, a cost-effective electrochemical sensor for the rapid and accurate determination of nitrate in aqueous solutions was developed. The sensor employs a tri-component SPE architecture (carbon black + electropolymerized poly 1,5-DAN + *in situ* Cu dendrites), a novel design that stabilizes Cu dendrites through polymer chelation and ensures reproducible dispersion, while carbon black provides efficient electron conduction. This mechanism produces uniform, reproducible catalytic sites rather than relying solely on morphology or exotic supports. Comprehensive electrochemical characterization was performed using CV and EIS techniques. Electrochemical studies showed that the synergistic combination of copper dendrites with poly 1,5-DAN and carbon black film significantly enhanced electron transfer and demonstrated excellent electrocatalytic activity for the sensing of nitrate ions. At the same time, XRD, XPS, FTIR, and SEM analyses confirmed the successful incorporation of Cu Ds into the poly 1,5-DAN/CB electrode. The fabricated sensor demonstrated high linearity, selectivity, and sensitivity, achieving a low LOD of 0.28 µM. Notably, the sensor enabled direct analysis of well water. Ongoing research is focused on further improving its performance and extending its applicability to broader environmental and clinical monitoring contexts.

## Author contributions

All authors listed have made a substantial, direct, and intellectual contribution to the work and approved it for publication.

## Conflicts of interest

The authors declare no conflicts of interest.

## Supplementary Material

RA-OLF-D6RA02725C-s001

## Data Availability

The data supporting this article, including raw electrochemical data, and data processing scripts, are available in the Zenodo repository at https://10.5281/zenodo.20618026. Additional supporting data have been included as part of the supplementary information (SI) File. The data used to support the findings of this study are included mainly in the article. Supplementary information is available. See DOI: https://doi.org/10.1039/d6ra02725c.
