# Supplementary material for: Rapid electrochemical nitrate sensing using flexible screen-printed electrodes modified with carbon black/poly (1,5-diaminonaphthalene)/copper dendrite hybrid interfaces
Source: RSC Adv. 2026 Jul 6;16(36):37473–91. doi: 10.1039/d6ra02725c (PMC13334451; doi:10.1039/d6ra02725c)
Supplement: Supplementary file 1 [file RA-016-D6RA02725C-s001.pdf]

Supplementary Information (SI) for RSC Advances.
This journal is © The Royal Society of Chemistry 2026

Rapid electrochemical nitrate sensing using flexible screen-printed electrodes modified with carbon black/ poly (1,5-diaminonaphthalene)/ copper dendrite hybrid interfaces

*Saad Benhaiba [a, b], Anas El Attar [c], Abdelaziz. Elgamouz [b], Charafeddine Jama [c], Amine Ezzahi [a], Mama El Rhazi [a]**

[a] *Laboratory of Materials Membranes and Environment, Faculty of Sciences and Technologies, University Hassan II Casablanca, BP 146, 20650 Mohammedia, Morocco*

[b] *Applied Chemistry Research Group, Department of Chemistry, College of Sciences, University of Sharjah, Sharjah, United Arab Emirates, P.O. Box 27272*

[c] *University Lille, CNRS, INRAE, Centrale Lille, UMR 8207-UMET-Unité Matériaux Et Transformations, 59000, Lille, France*

*mama.elrhazi@fstm.ac.ma

Corresponding author:
Mama El RHAZI (Faculty of Sciences and Technologies – BP 146 Mohammedia 20650, University Hassan II of Casablanca, Morocco)
mama.elrhazi@fstm.ac.ma
Tel: 212 523315352 Fax: 212 523315353

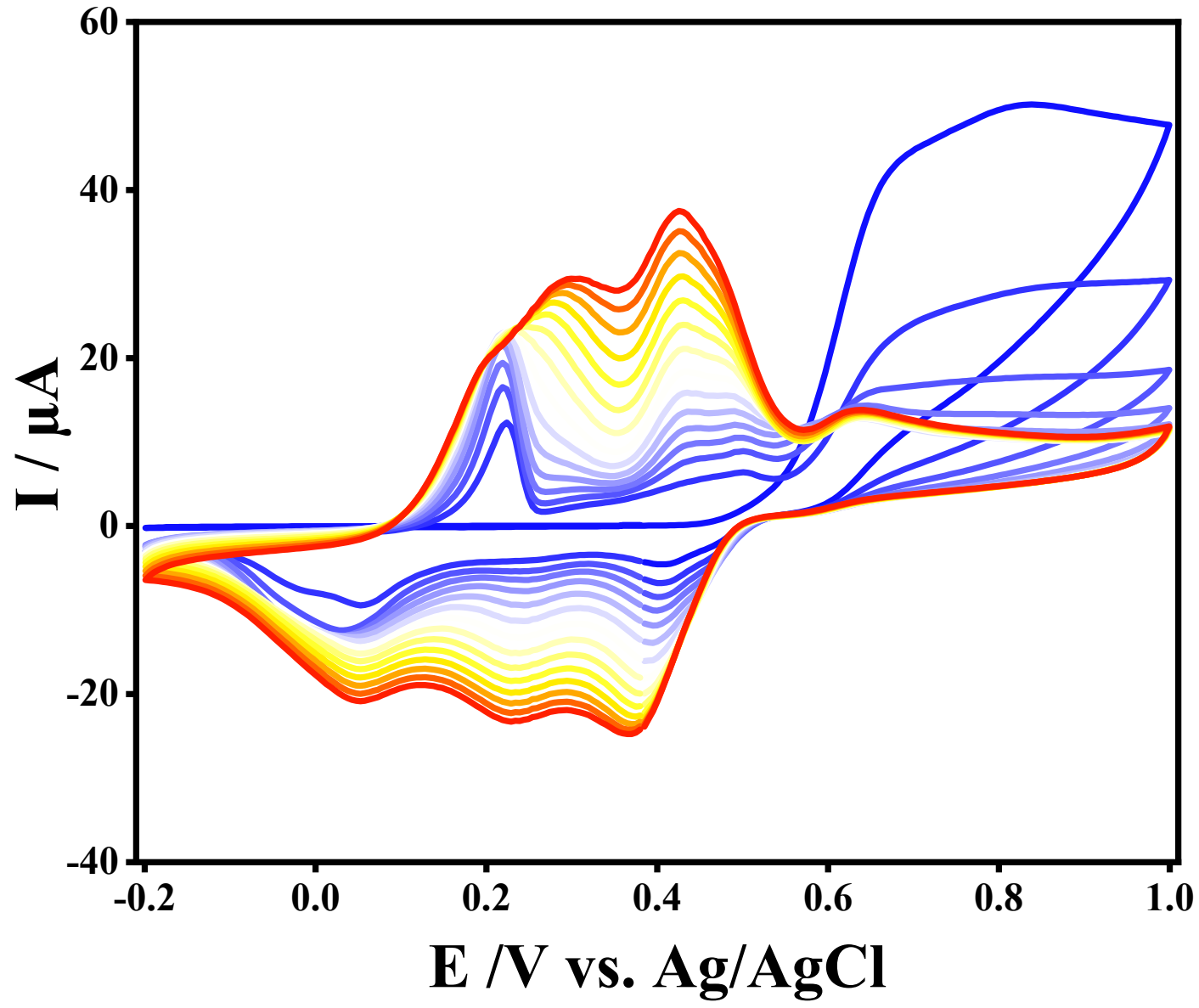

Fig.S1. CV of SPE in 0.1 M HCl containing 5 mM of 1,5-DAN for 15 cycles at a scan rate of 50 mV/s.

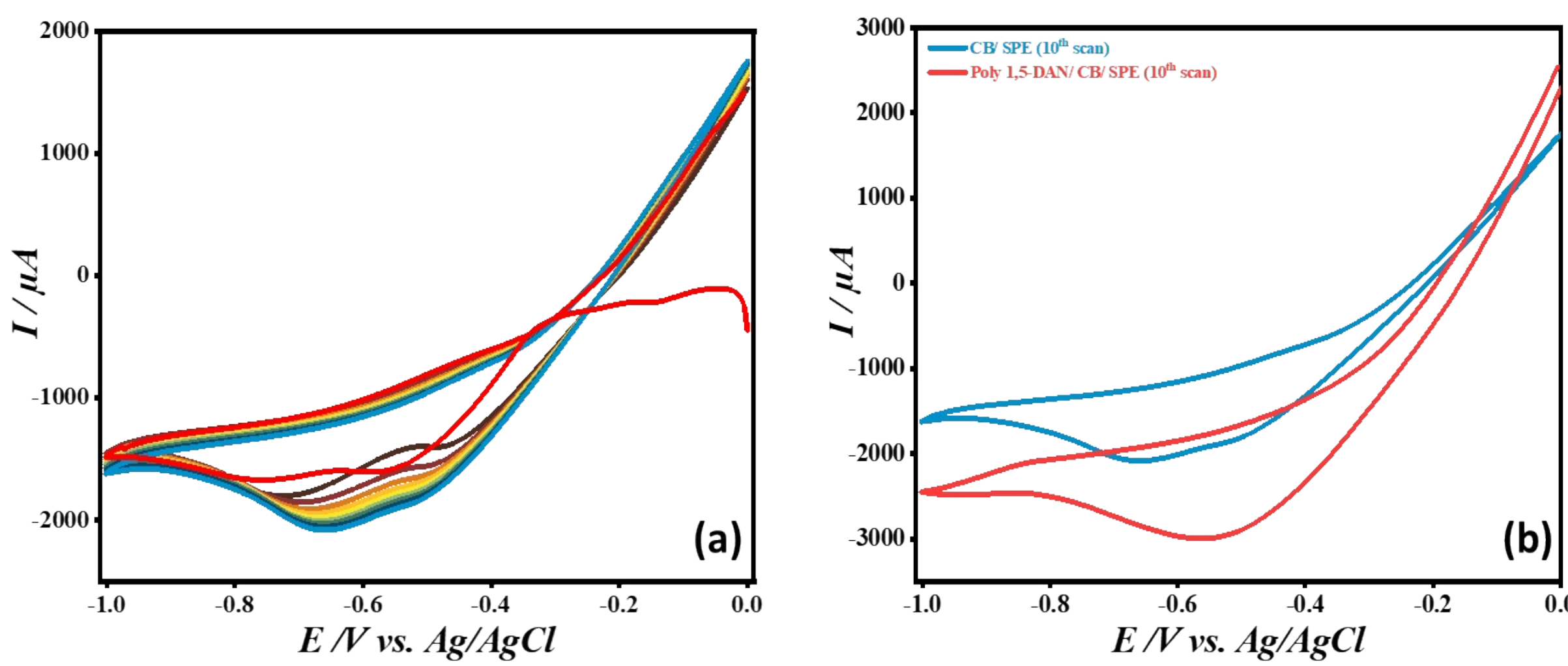

Fig.S2. (a) Electrodeposition of copper on CB/SPE using CV in a solution of 0.1 M H_2SO_4 containing 0.1 M $CuSO_4$, (b) 10th scan of the electrodeposition of copper on CB/SPE and Poly 1,5-DAN/ CB/ SPE

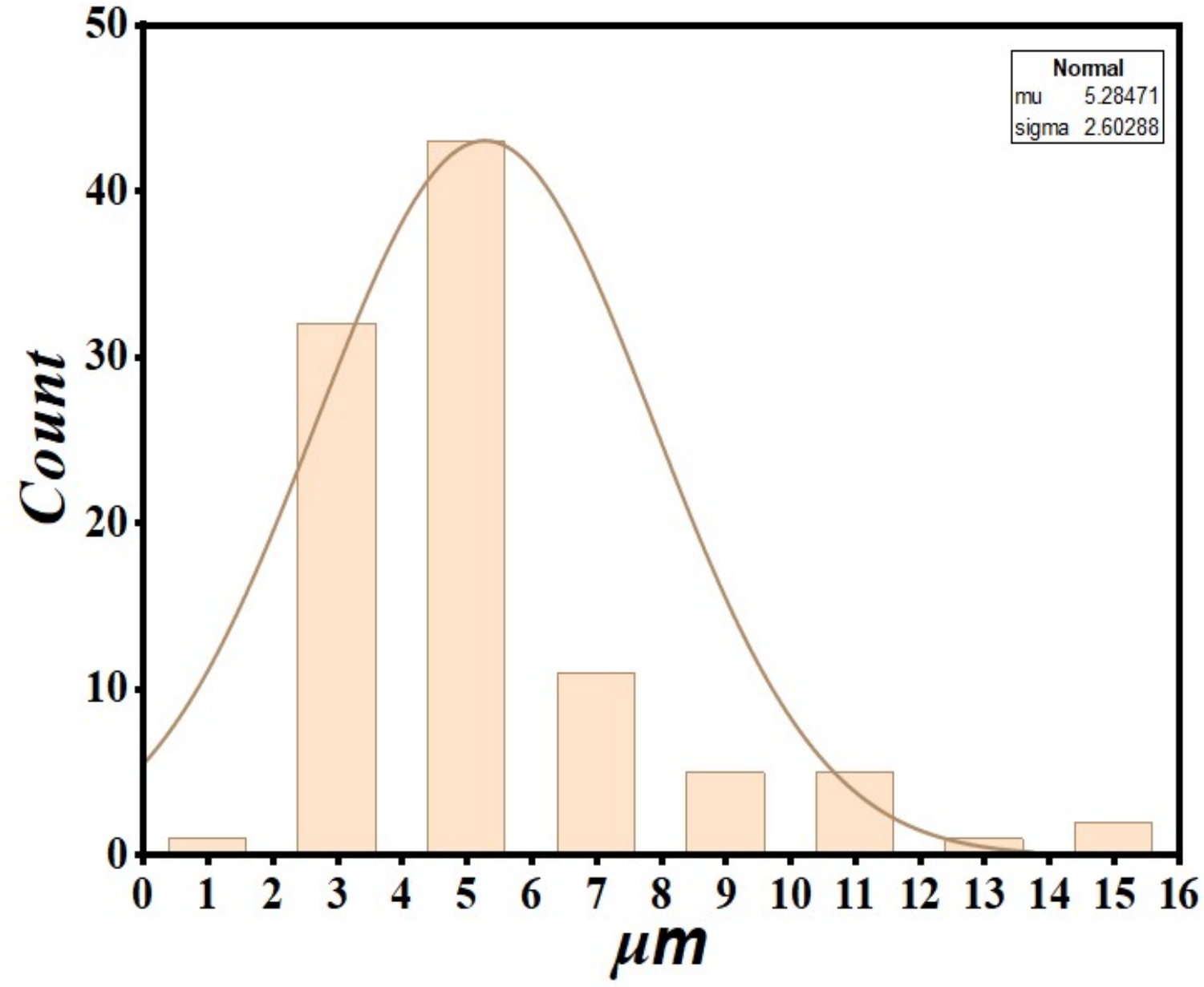

Fig. S3. Distribution size of the copper dendrites /100 particles.

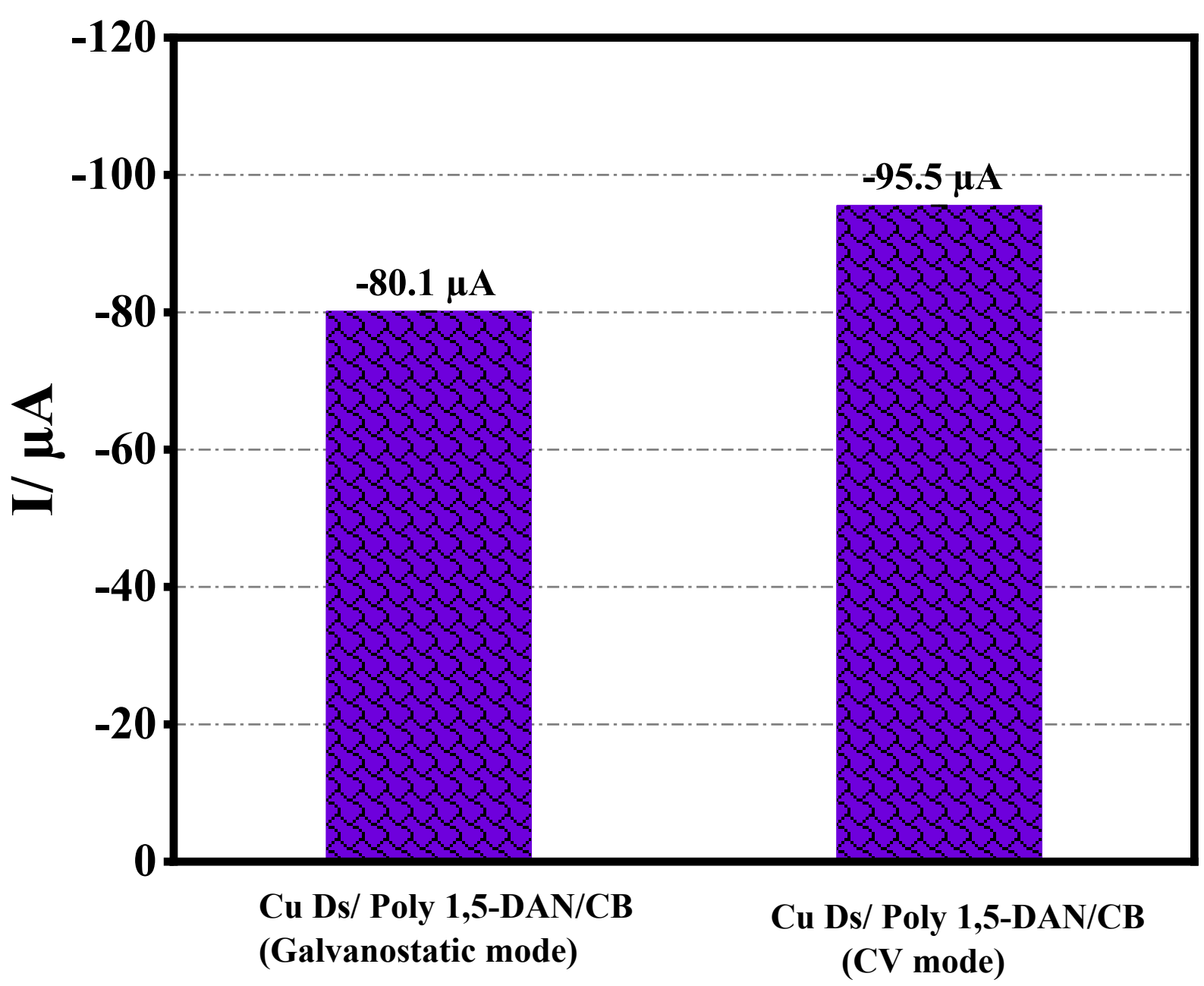

Fig. S4. Effect of the electropolymerization mode of Poly-1,5-DAN on the response current toward NO_3^-.

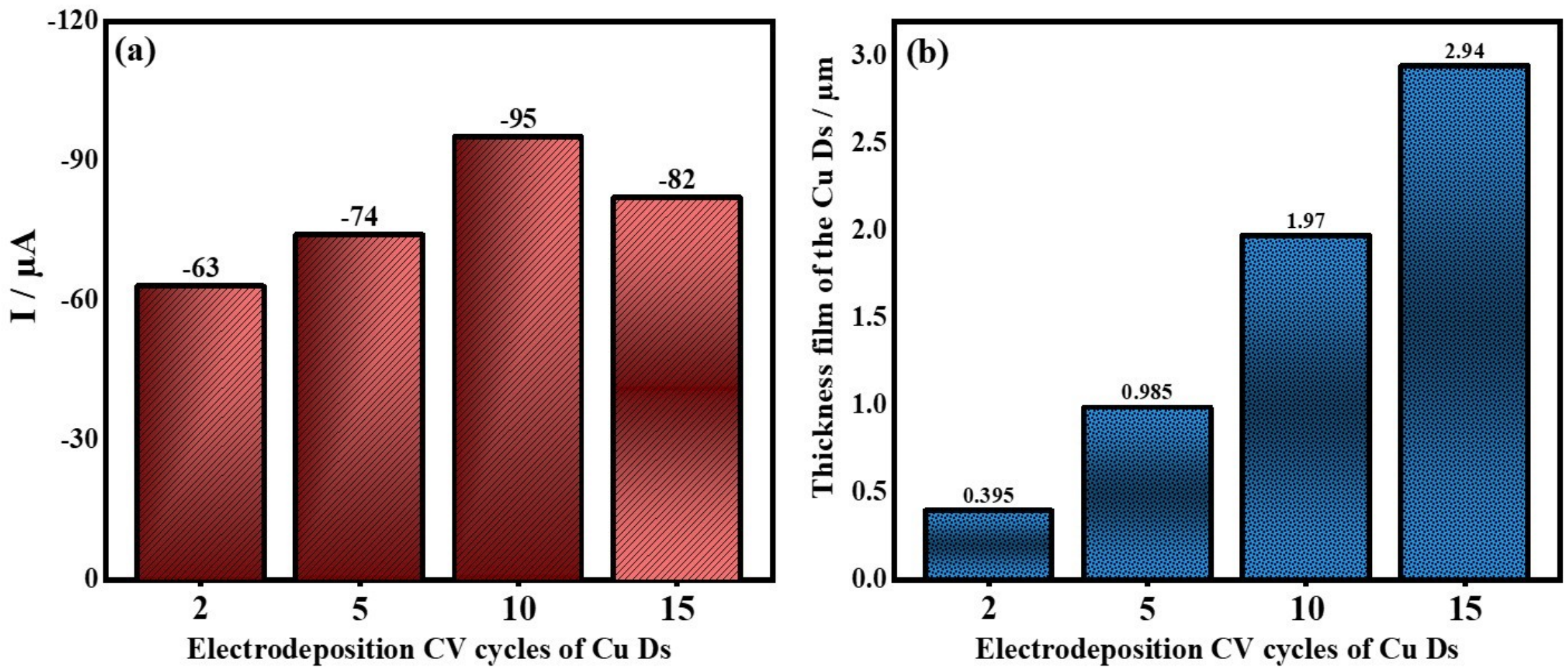

Fig. S5. (a) Reduction peak current of the nitrate sensor (1.0 mM NO_3^-) prepared using different numbers of CV cycles during Cu electrodeposition. (b) Thickness film of the Cu Ds versus electrodeposition CV cycles.

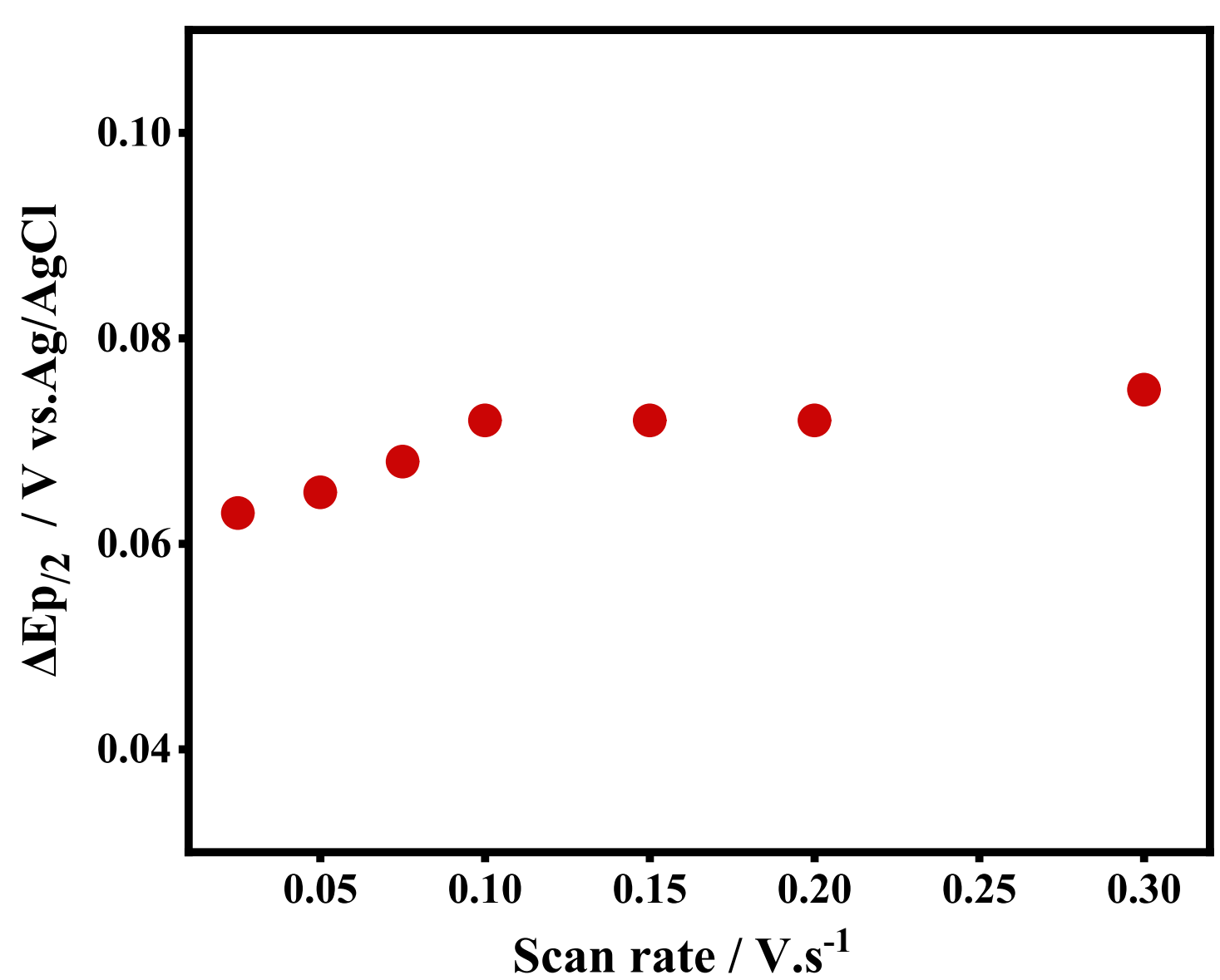

Fig. S6 Scan rate dependency of $\Delta E_{p/2}$ for the nitrate reduction

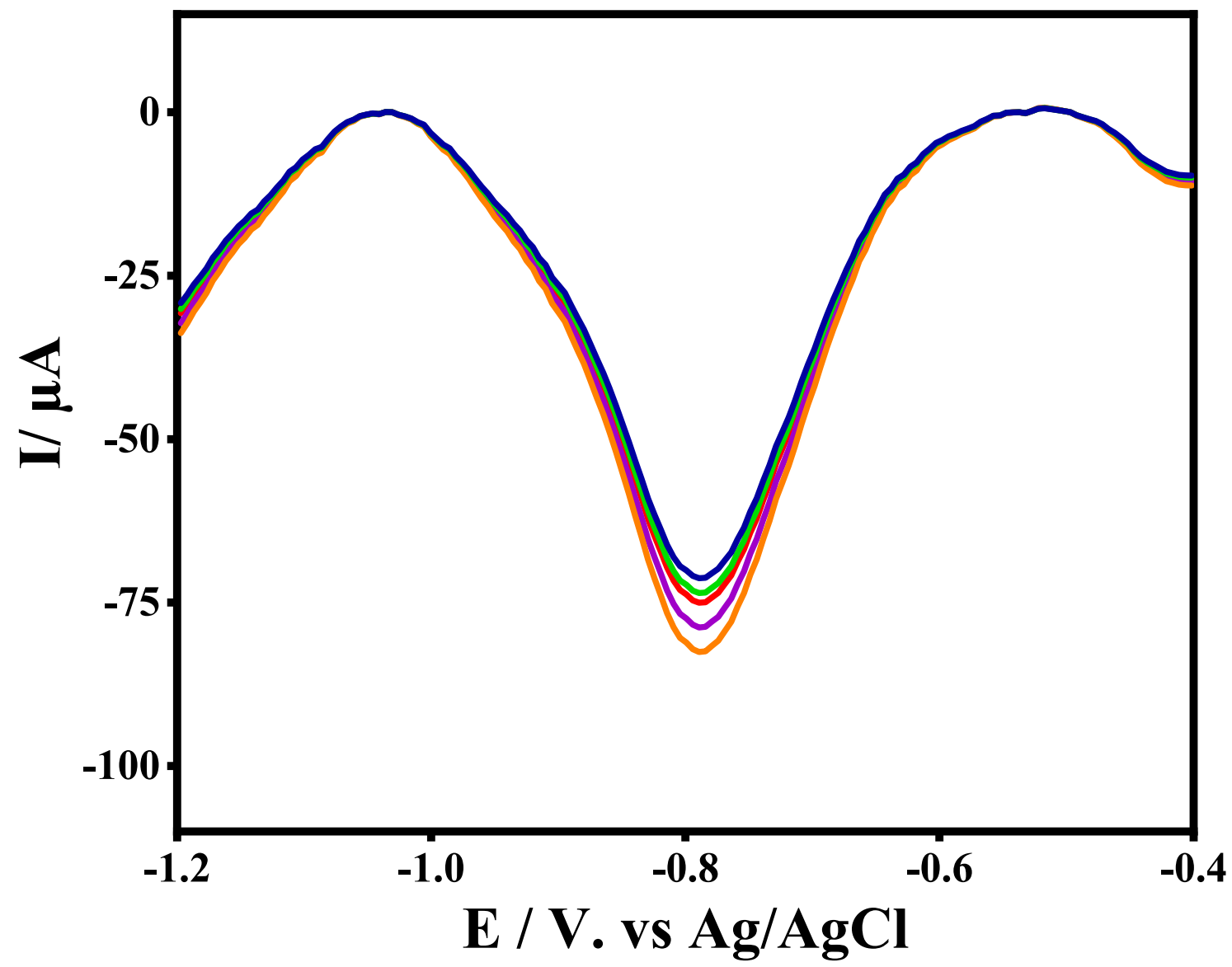

Fig. S7 SWV of the repeatability of the Cu Ds/Poly(1,5-DAN)/CB/SPE in C=100 µM of NO_3^-

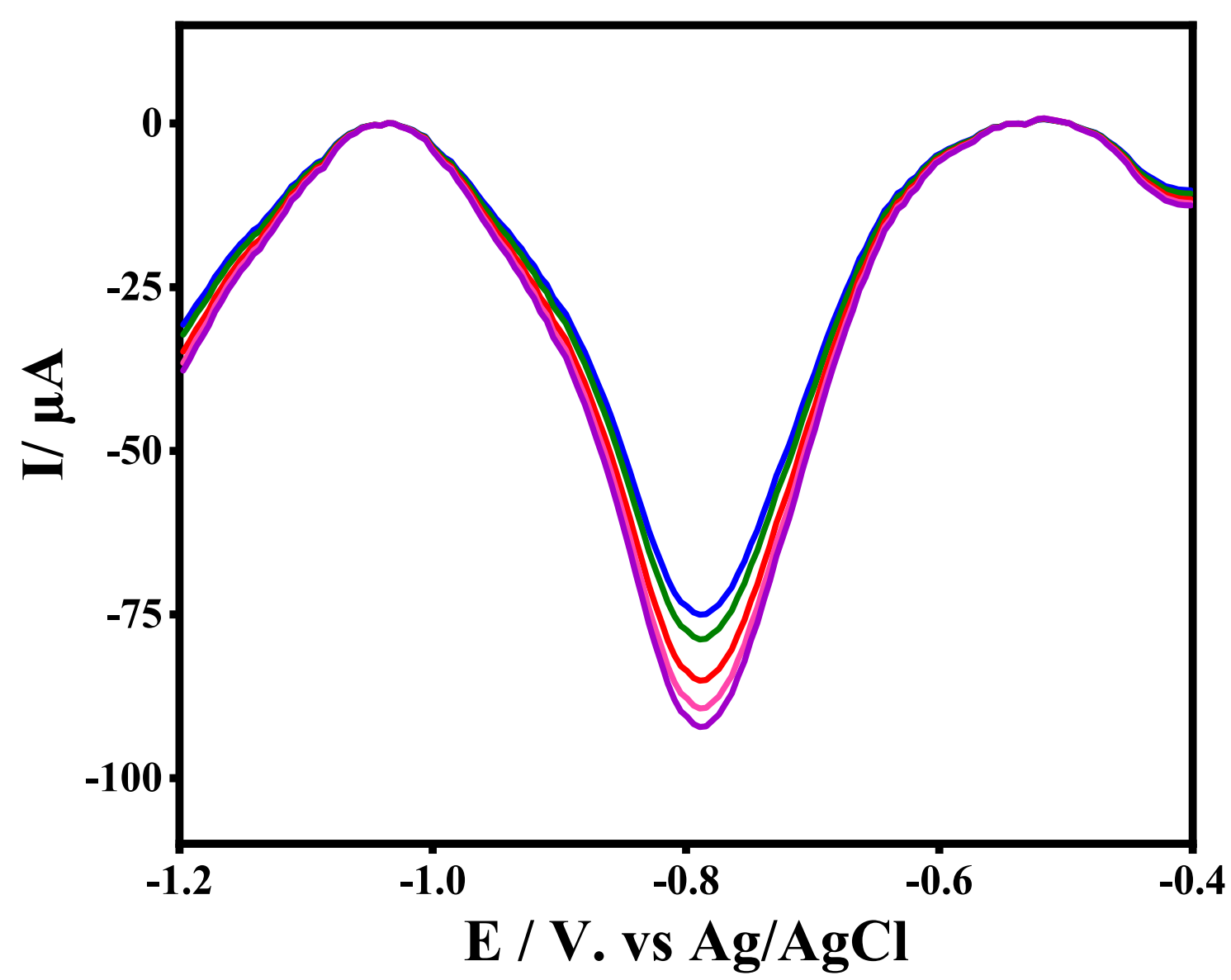

Fig. S8 SWV of the reproducibility of the Cu Ds/Poly(1,5-DAN)/CB/SPE in C=100 µM of NO_3^-

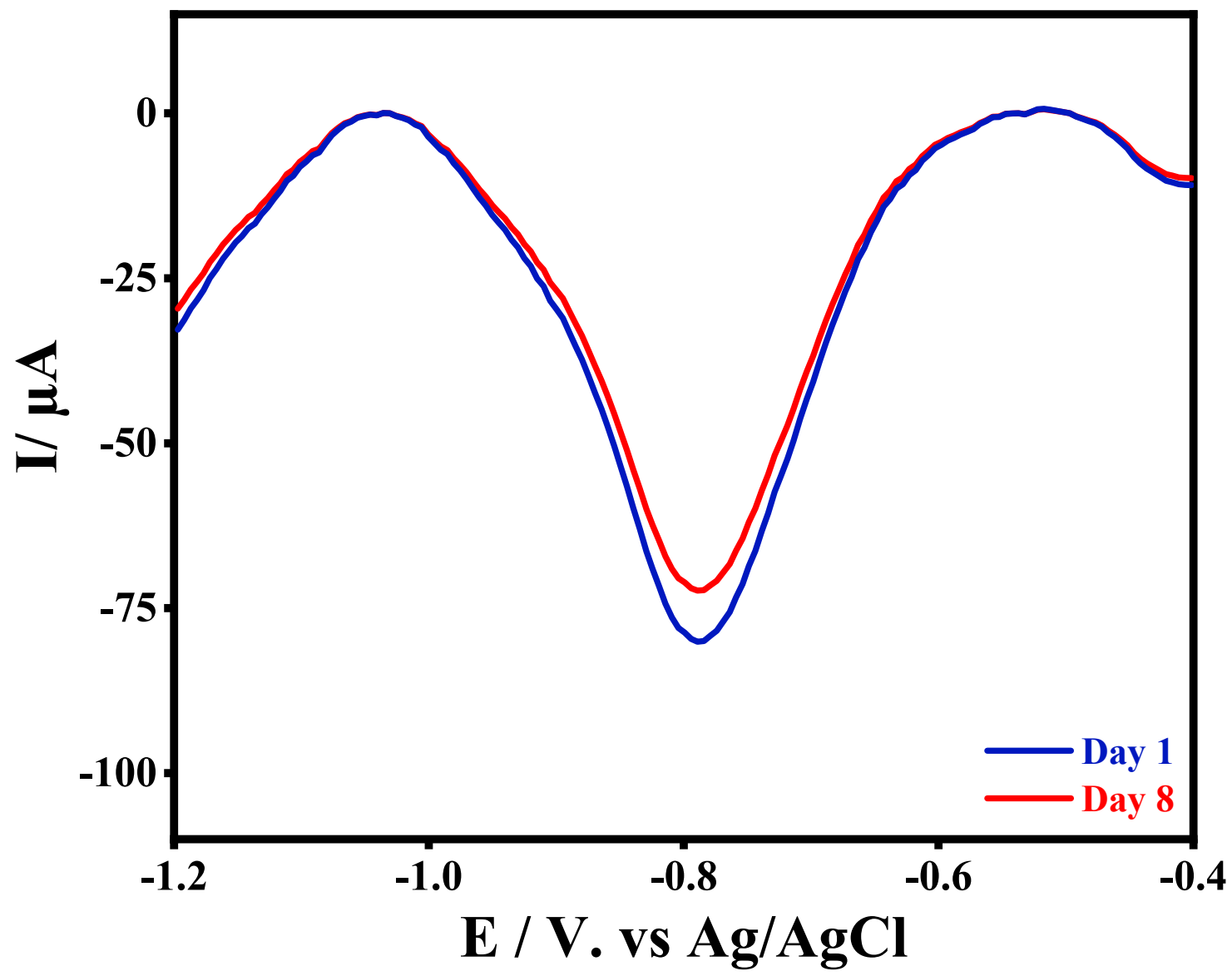

Fig. S9 SWV of the stability of the Cu Ds/Poly(1,5-DAN)/CB/SPE in C=100 µM of NO_3^-
